# Structure and functionality in flavivirus NS-proteins: Perspectives for drug design

**DOI:** 10.1016/j.antiviral.2009.11.009

**Published:** 2010-08

**Authors:** Michela Bollati, Karin Alvarez, René Assenberg, Cécile Baronti, Bruno Canard, Shelley Cook, Bruno Coutard, Etienne Decroly, Xavier de Lamballerie, Ernest A. Gould, Gilda Grard, Jonathan M. Grimes, Rolf Hilgenfeld, Anna M. Jansson, Hélène Malet, Erika J. Mancini, Eloise Mastrangelo, Andrea Mattevi, Mario Milani, Grégory Moureau, Johan Neyts, Raymond J. Owens, Jingshan Ren, Barbara Selisko, Silvia Speroni, Holger Steuber, David I. Stuart, Torsten Unge, Martino Bolognesi

**Affiliations:** a Department of Biomolecular Sciences and Biotechnology, University of Milano, Via Celoria 26, 20133 Milano, Italy; b Laboratoire Architecture et Fonction des Macromolécules Biologiques, CNRS UMR-6098, Universités Aix-Marseille I et II, ESIL Case 925, 163 Avenue de Luminy, 13288 Marseille, France; c Oxford Protein Production Facility, Wellcome Trust Centre for Human Genetics, Roosevelt Drive, Headington, Oxford OX3 7BN, UK; d Unité des Virus Emergents, Faculté de Médecine, 27 Bd Jean Moulin, 13005 Marseille, France; e The Natural History Museum, Cromwell Road, London, United Kingdom; f Centre for Ecology and Hydrology, Mansfield Road, Oxford OX1 3SR, United Kingdom; g Institute of Biochemistry, Center for Structural and Cell Biology in Medicine, University of Lübeck, Ratzeburger Allee 160, 23538 Lübeck, Germany; h Department of Cell and Molecular Biology, Uppsala University, Biomedical Center, Box 596, SE-751 24 Uppsala, Sweden; i CNR-INFM S3, National Research Center on Nanostructure and BioSystems at Surfaces, Via Campi 213/A, 41100 Modena, Italy; j Department of Genetics and Microbiology, University of Pavia, Via Ferrata 7, 27100 Pavia, Italy; k Rega Institute for Medical Research, KULeuven - University of Leuven, Minderbroedersstraat 10, 3000 Leuven, Belgium

**Keywords:** BVDV, bovine viral diarrhea virus, C, capsid protein, CSFV, classical swine fever virus, CCHFV, Crimean-Congo hemorrhagic fever virus, CPE, cyto-pathogenic effect, dsRNA, double-stranded RNA, ER, endoplasmic reticulum, E, envelope protein, GMP, guanosine monophosphate, GTP, guanosine triphosphate, GTase, guanylyltransferase, NS3Hel, helicase, HIV, Human Immunodeficiency Virus I, HCV, hepatitis C virus, HBS, high affinity binding site, IMP, Inosine 5′-monophosphate, LBS, low-affinity binding site, M, membrane protein, NS5MTase, methyltransferase, N7MTase, (guanine-N7)-methyltransferase, 2′OMTase, (nucleoside-2′-O-)-methyltransferase, NS, non-structural, NLS, nuclear localization sequences, NS3Pro, protease, RC, replication-competent complex, RSV, respiratory syncytial virus, NS5RdRp, RNA-dependent RNA polymerase, NS3RTPase, RNA triphosphatase, AdoMet, S-adenosyl-L-methionine, ssRNA, single-stranded RNA, T-705 RMP, T-705-ribofuranosyl-5′-monophosphate, VIZIER, Viral Enzymes Involved in Replication, Flavivirus, Flaviviral NS3 protein, Flaviviral NS5 protein, Protease, Helicase, Polymerase, Methyltransferase, Flavivirus protein structure, Antivirals, VIZIER Consortium

## Abstract

Flaviviridae are small enveloped viruses hosting a positive-sense single-stranded RNA genome. Besides yellow fever virus, a landmark case in the history of virology, members of the Flavivirus genus, such as West Nile virus and dengue virus, are increasingly gaining attention due to their re-emergence and incidence in different areas of the world. Additional environmental and demographic considerations suggest that novel or known flaviviruses will continue to emerge in the future. Nevertheless, up to few years ago flaviviruses were considered low interest candidates for drug design. At the start of the European Union VIZIER Project, in 2004, just two crystal structures of protein domains from the flaviviral replication machinery were known. Such pioneering studies, however, indicated the flaviviral replication complex as a promising target for the development of antiviral compounds. Here we review structural and functional aspects emerging from the characterization of two main components (NS3 and NS5 proteins) of the flavivirus replication complex. Most of the reviewed results were achieved within the European Union VIZIER Project, and cover topics that span from viral genomics to structural biology and inhibition mechanisms. The ultimate aim of the reported approaches is to shed light on the design and development of antiviral drug leads.

## 1 Introduction

The genus Flavivirus, together with Pestivirus and Hepacivirus, belongs to the family of Flaviviradae. Flaviviridae are small enveloped viruses hosting a positive-sense single-stranded RNA genome. The complete genome is 9500–12,500 nucleotides long. It encodes a large polyprotein precursor, which is co- and post-translationally processed by viral and cellular proteases into three structural proteins, building the capsid, and seven non-structural proteins involved in virus replication.

### 1.1 Emergence and re-emergence of pathogenic flaviviruses

In the Flaviviridae family, the genus Flavivirus occupies a special space within the RNA virus world. The family derives its name from the word flavus (Latin for yellow), with one prominent member being the yellow fever virus (YFV) a landmark reference system in the history of virology. It was introduced in the Americas in the 16th century as a consequence of the African slave trade, recognized by Carlos Finlay as a vector-borne disease as early as 1881, before any virus was isolated. YFV was the first human pathogenic virus isolated in 1927 (Staples and Monath, 2008). Although a safe and efficient vaccine designed in 1937 by Max Theiler shaped our view on the control of viruses, there are still more than 200,000 annual cases in Africa alone, and about 15% of the cases enter a critical phase that only 50% of the patients survive (Ellis and Barrett, 2008). In more recent years, members of the Flavivirus genus gained public visibility due to re-emergence and steadily increasing incidence, such as for West Nile virus (WNV) in the Americas and dengue virus (DENV) in subtropical areas of the world.

WNV, isolated in Uganda in 1937, is endemic in Africa and southern Europe, but its appearance in the Americas in 1999 was followed by a rapid geographic extension from Canada to Argentina by 2008, leaving behind thousands of deaths and disabled patients (Petersen and Hayes, 2008). Likewise, the four DENV serotypes have considerably expanded their geographic distribution in recent years. With billions of people at risk, more than 50 million cases, and about 12,500–25,000 deaths annually, DENV is robustly emerging in a growing number of countries (Vasilakis and Weaver, 2008). The two remaining clinically significant flaviviruses are the Japanese encephalitis virus (JEV) and tick-borne encephalitis virus (TBEV), for which existing vaccines should help reduce the current morbidity burden, mostly in Asia and central Europe, respectively. Most flaviviruses are arthropod-borne viruses (arboviruses), transmitted either by ticks (tick-borne viruses, TBV) or mosquitoes (mosquito-borne viruses, MBV), but a number of flaviviruses have no known vectors (NKV) and/or have been isolated from infected animals without a link to any specific disease (Table 1).

**Table 1:** Flaviviral abbreviation.

Tick-borne viruses	TBVs
Gadget Gully virus	GGYV
Kadam virus	KADV
Kyasanur Forrest disease virus	KFDV
Langat virus	LGTV
Omsk hemorrhagic fever virus	OHFV
Powassan virus	POWV
Royal farm virus	RFV
Karshi virus	KSIV
tick-borne encephalitis virus	TBEV
Louping ill virus	LIV
Meaban virus	MEAV
Saumarez Reef virus	SREV
Tyuleniy virus	TYUV
Ngoye virus	NGOV

Mosquito-borne viruses	MBVs
Aroa virus	AROAV
Bussuquara virus	BSQV
Iguape virus	IGUV
Naranjal virus	NJLV
Dengue virus	DENV
Kedougou virus	KEDV
Cacipacore virus	CPCV
Koutango virus	KOUV
Japanese encephalitis virus	JEV
Murray Valley encephalitis virus	MVEV
Alfuy virus	ALFV
St Louis encephalitis virus	SLEV
Usutu virus	USUV
West Nile virus	WNV
Kunjin virus	KUNV
Yaounde virus	YAOV
Kokobera virus	KOKV
Stratford virus	STRV
Bagaza virus	BAGV
Ilheus virus	ILHV
Rocio virus	ROC
Israel turkey meningoencephalomyelitis virus	ITV
Ntaya virus	NTAV
Tembuzu virus	TMUV
Zika virus	ZIKV
Spondweni virus	SPOV
Banzi virus	BANV
Bouboui virus	BOUV
Edge Hill virus	EHV
Jugra virus	JUGV
Potiskum virus	POTV
Saboya virus	SABV
Sepik virus	SEPV
Uganda S virus	UGSV
Sitiawan virus	SV
Kamiti River virus	KRV
Wesselsbron virus	WESSV
Yellow fever virus	YFV
Nounané virus	NOUV
Barkedji virus	

Viruses with no known arthropod vector	NKVs
Entebbe bat virus	ENTV
Sokoluk virus	SOKV
Yokose virus	YOKV
Apoi virus	APOIV
Cowbone Ridge virus	CRV
Jutiapa virus	JUTV
Modoc virus	MODV
Sal Vieja virus	SVV
San Perlita virus	SPV
Bukalasa bat virus	BBV
Carey Island virus	CIV
Dakar bat virus	DAKV
Montana Myotis leukoencephalitis virus	MMLV
Phnom Penh bat virus	PPBV
Batu Cave virus	BCV
Rio Bravo virus	RBV
Cell fusing agent virus	CFAV
Tamana bat virus	TABV

### 1.2 Development of flavivirus treatments

There are a number of environmental, demographic and ecological reasons to believe that either novel or known flaviviruses will continue to emerge. In this respect, the success of vaccination against YFV has been temperated by difficulties encountered when such programs were launched against DENV. In particular, the presence of four DENV serotypes has complicated vaccine design because incomplete protection against one serotype may influence the disease outcome once infection is established by a distinct serotype, through a process referred to as antibody-mediated disease enhancement (Guzman and Kouri, 2008). Therefore, in addition to vaccine design efforts, there has been a growing interest in discovering drugs against DENV and WNV. For instance, a moderate, borderline effect, whose mechanism of action is controversial, was reported for the activity of ribavirin against flaviviruses (Huggins, 1989, Day et al., 2005, Leyssen et al., 2006, Takhampunya et al., 2006). Prior to 2004 there were very few coordinated efforts towards the design of antiflavivirus compounds, flaviviruses being hardly considered interesting candidates for drug design. A notable exception has been the activity at the Novartis Institute for Tropical Disease in Singapore that focused its research efforts on dengue disease since its first opening (in 2003) (Gubler and Clark, 1995, Kroeger et al., 2004). Perhaps even before the launch of the European Union VIZIER Project (Viral Enzymes Involved in Replication) in October 2004, the lack of viral genomics programs was recognized as a problem for any research activity aiming at the discovery and design of antiviral drugs based on crystal structure information. Indeed, since a single amino acid substitution can determine resistance to a given drug, systematic benchmarking of starting genetic material and resulting data was highly sought after. Few complete flavivirus genome sequences were known at the launch of VIZIER (less than 30 out of >70), and the commitment to sequence the entire Flavivirus genus in VIZIER (see below) was of key importance in the standardization of cDNA targets and their referencing during the project.

### 1.3 Molecular biology of flavivirus polyprotein processing and replication: the roles of NS5 and NS3

The ∼11 kb flavivirus RNA genome is a positive-sense, single-stranded, 5′-capped RNA ((+)ssRNA) that is released into the cytoplasm immediately following cell entry. It encodes a single, large polyprotein, which is proteolytically processed to yield three structural proteins (envelope, E; membrane precursor, PrM; and capsid C) and seven non-structural (NS) proteins (NS1, NS2a, NS2b, NS3, NS4a, NS4b, and NS5). The polyprotein is cleaved co- and post-translationally by a combination of cellular proteases of the furin-type or other Golgi-localized proteases and the viral serine protease embedded in the N-terminal domain of non-structural protein 3 (NS3Pro), which requires NS2B for its activity. NS proteins are thought to co-translationally assemble on the endoplasmic reticulum (ER) membranes forming the replication competent complex, which consists morphologically distinct, membrane-bound compartments that also differ with respect to both function and NS proteins composition (reviewed in: Mackenzie, 2005). The NS3 and NS5 proteins are central to the viral RC, as together they harbour most, if not all, of the catalytic activities required to both cap and replicate the viral RNA. NS3 is a multidomain protein, with an N-terminal NS3Pro as discussed above, and a C-terminal portion containing the RNA triphosphatase (NS3RTPase) and RNA helicase (NS3Hel) activities involved in capping and viral RNA synthesis, respectively. NS5 consists of an N-terminal methyltransferase (NS5MTase) domain and the C-terminal RNA-dependent RNA polymerase (NS5RdRp) domain. During these processes, the (+)ssRNA viral genome acts as a template for: (1) the synthesis of the intermediate (−)ssRNA strand by the NS5 RdRp, which in turn acts as template solely for the synthesis of (+)ssRNA genomic RNAs (again by the NS5), and (2) the synthesis of the viral polyprotein.

The 5′-end of the (+)ssRNA genome is decorated by a RNA cap structure (N7meGpppA2′Ome-RNA). It plays an essential role, acting, as for eukaryotic mRNAs, to initiate the process of translation and to protect the viral RNA from degradation by endogenous RNA exonucleases. It is also a unique feature of the flavivirus genome in the context of the Flaviviridae family as a whole, since pesti- and hepaciviruses do not possess it. In flaviviruses, mRNA capping is thought to start with the conversion of the 5′-triphosphate mRNA into a diphosphate by the RNA triphosphatase domain embedded in the C-terminal domain of the NS3 protein (NS3RTPase). The second reaction is the transfer of a guanosine monophosphate (GMP) moiety from a guanosine triphosphate (GTP) to 5′-diphosphate RNA, to yield G5′-ppp-N, by a guanylyltransferase (GTase). Afterwards, the transferred guanosine moiety is methylated by the N-terminal methyltransferase domain of the NS5 protein (NS5MTase). To date, the molecular species responsible for the GTase activity remains unknown (Ahola and Kaariainen, 1995, Furuichi and Shatkin, 2000, Egloff et al., 2002, Ray et al., 2006), although recent evidences suggest that it might be associated to the NS5MTase domain (Egloff et al., 2007, Bollati et al., 2009b).

Although the details of flavivirus replication have seen major advances in the past years, many aspects remain not fully understood. For instance, an increasing number of studies have shown that specific RNA structures present in the 5′ and 3′ UTR regions play a critical role in replication and capping, with genome cyclization being one of several processes identified on which replication depends. However, the precise details, such as how NS5 and/or NS3Hel activities might be controlled by such structures, remain to be established. Equally, the role of NS3Hel in these processes remains to be established formally and hence, analyzed in more detail, although it is thought to be at least involved in the formation of the 5′ cap structure of viral RNA and in the unwinding of dsRNA intermediates that arise during replication. Observations such as those showing that NS3Hel has an apparently unrelated function in the downstream assembly of the virion indicate that many aspects of NS3 as well as NS5 function remain to be established.

Following replication the protected, genomic RNA is packaged by the C protein in a host-derived lipid bilayer in which the E protein is embedded. The mature particles subsequently exit from the host cell by exocytosis.

### 1.4 The VIZIER context

At the start of the VIZIER Project, crystal structures of only two flavivirus replication protein domains (DENV NS3Pro, Murthy et al., 1999; and the DENV NS5MTase, Egloff et al., 2002) were known. In addition to their biological relevance (discussed below) these studies had a pioneering value since they showed that individual domains of NS3 and NS5 could be produced in isolation and their crystal structure solved.

As a result, flavivirus NS3 and NS5 proteins were held as targets for the VIZIER Project. On one hand, NS3 and NS5 constitute important drug targets, and on the other they were held s targets within reach for large scale production and crystallization, thereby facilitating the cementing of the VIZIER community, the beta-testing of the communication protocols and project pipelines, and the establishment of bridges between the structural biology and virology laboratories expertises.

In the following sections of this paper, we will present the collective efforts developed for the characterization of several flavivirus molecular aspects within the VIZIER Project (http://www.vizier-europe.org/), from viral genomics to structural biology approaches focused on flavivirus NS3 and NS5, emphasizing the implications that the data produced bear for antiviral drug development.

## 2 Flavivirus genomics

The flaviviruses comprise a fascinating group of viruses, occupying a very special position in the history of virology due to their taxonomic, epidemiological and pathogenetic characteristics, which include the following:

(1) The genus Flavivirus, contains an unusually large number of viruses (more than 70), that are distributed globally. The genus also includes a large, and increasing, number of unclassified or “tentative” species that have very different characteristics from those currently recognized as members of the genus.
(2) Among the flaviviruses there are more than 40 human pathogens, responsible for a variety of diseases ranging from poorly specific pseudo-flu-like syndromes, to severe encephalitic or hemorrhagic disease. One flaviviral disease of particular note is dengue fever, which is estimated to cause in excess of 50 million cases per year (WHO, Fact sheet No. 117, March 2009). Many other flaviviral diseases, such as West Nile fever, Japanese encephalitis, and Zika fever are classified as emerging diseases.
(3) YFV is the prototype species of the genus flavivirus. The 17D vaccine is one of the most efficient vaccines ever developed and was derived from a strain of YFV isolated from a man who recovered from infection by the virus.
(4) Flaviviruses are “complex” viruses, with various – and poorly understood – ecological cycles. Importantly, most of the human pathogens are transmitted by arthropods (i.e. they are “arboviruses”). However, viruses with no known vector, or viruses that infect only arthropods (tentatively referred to as “insect-only” flaviviruses) have also been identified. This remarkable diversity is associated with broad genetic variability and complex mechanisms of pathogenesis.
(5) The genus flavivirus underlines the history of mammalian virology since the first human viral pathogen to be demonstrated as a filterable agent was YFV (Reed, 1901a, Reed, 1901b) and subsequently the first human viral pathogens isolated experimentally were YFV (Theiler, 1930) and Louping ill virus (LIV) (Greig et al., 1931), followed soon after by DENV and African horse sickness virus.
(6) Flaviviruses have historically been associated with changes in taxonomy that reflected advances in virology. Firstly, the term arborviruses (subsequently changed to arboviruses to avoid confusion with the Latin word “arbor”, meaning tree) was derived as a taxonomic criterion following the discovery of several arthropod-borne viruses. Subsequently, morphological information obtained using electron microscopy supported the hypothesis of the existence of at least two virus groups:

• One group includes non-enveloped viruses, which currently are classified within the family Reoviridae (genera Orbivirus, Coltivirus and Seadornavirus), i.e. viruses with an overall diameter of 60–80 nm, icosahedral symmetry and several concentric capsid layers that surround a segmented double-stranded RNA (dsRNA) genome.
• A second group includes enveloped viruses (inactivated by ether and deoxycholate), 50–60 nm in diameter, with an infectious ssRNA genome of positive polarity. The development of serological methods led to the identification of two antigenically distinct sub-groups. This division was subsequently confirmed by analysis of the genome sequences and the viruses were divided as follows:
  (a) The “Group A arboviruses”, comprising viruses currently classified within the genus Alphavirus, family Togaviridae (together with the non-arboviral genus Rubivirus).
  (b) The “Group B arboviruses”, comprising viruses currently classified within the genus Flavivirus, family Flaviviridae (together with non-arboviral genera Hepacivirus and Pestivirus).

### 2.1 The first steps in flavivirus genomics

The history of flavivirus genomics did not start with the progressive accumulation of partial genome sequences but, surprisingly, with the publication in 1985 of a seminal study by Rice et al. (1985) who determined the complete genome sequence of YFV. The work of Rice and his collaborators was remarkable because it unexpectedly established that the flavivirus genome strategy was very distinct from that of the alphaviruses that had been grouped taxonomically in the same virus family. Indeed, results demonstrated the existence of 5′ and 3′ non-coding regions and, a unique single open reading frame that encoded a polyprotein containing all the structural proteins in the N-terminal region of the genome and all the non-structural proteins in the C terminal region of the genome.

This founding discovery was followed by the rapid characterization of a large number of complete sequences for other flaviviruses: WNV (Castle et al., 1986), JEV (Sumiyoshi et al., 1987), Kunjin virus (KUNV) (Coia et al., 1988), DENV4 (Zhao et al., 1986, Mackow et al., 1987), DENV2 (Hahn et al., 1988, Irie et al., 1989), TBEV (Mandl et al., 1989, Pletnev et al., 1990), DENV3 (Osatomi and Sumiyoshi, 1990), DENV1 (Fu et al., 1992), Powassan virus (POWV) (Mandl et al., 1993), LIV (Gritsun et al., 1997), Murray Valley encephalitis virus (MVEV) (Hurrelbrink et al., 1999), and Langat virus (LGTV) (Campbell and Pletnev, 2000).

This first series of full-length genome sequences included the first “atypical” flavivirus. In 1992, Cammisa-Parks et al. (1992) reported the discovery and complete characterization of Cell-Fusing Agent virus (CFAV). For the first time, a very distantly related virus was studied and, importantly, results implied that the flavivirus lineage included viruses which infect only mosquitoes, in other words they are insect viruses which do not appear to infect mammals. Together with the previous isolation and antigenic characterization of a number of viruses with no identified vector (i.e. infecting only vertebrates) such as Rio Bravo virus (RBV) (Burns et al., 1957, Johnson, 1957), this provided robust evidence that the ecological and genetic complexity of the flaviviruses had been under-estimated.

### 2.2 E gene and NS5 datasets

In parallel, studies of partial sequences commenced, focusing mainly on flavivirus E genes. Increased availability of E gene data enabled the construction of the first robust phylogenies for the genus. Importantly, these studies globally confirmed the previous classification of flaviviruses (Porterfield, 1980, Calisher et al., 1989) based on antigenic relationships (Blok et al., 1992, Lewis et al., 1993, Mandl et al., 1993, Zanotto et al., 1996), but also established milestone observations regarding flaviviral evolution. In particular, they suggested that TBVs and MBVs evolved independently from a common ancestor, that viruses belonging to the tick-borne encephalitis complex evolved as an arboviral cline across the northern hemisphere, and that, within the group of MBVs, the lineage of *Culex* spp.-associated flaviviruses emerged from that of *Aedes* spp. associated viruses.

In 1998, Kuno et al. (1998) published a genetic study based on partial NS5 RdRp sequences. For the first time, phylogenies included a very large number of flaviviruses from different genetic or ecological groups, i.e. MBVs and TBVs, also in addition to NKVs, plus CFAV. This study confirmed the major findings of previous E gene phylogenies, but also led to clarification of the two different groups of NKV: one that constitutes a large independent lineage (e.g. RBV, Apoi virus (APOIV), Bukalasa bat virus (BBV), Modoc virus (MODV), etc.) and one that is related to YFV, within the group of Aedes-borne viruses (Entebbe bat (ENTV), Yokose (YOKV) and Sokuluk (SOKV) viruses).

### 2.3 Recent advances in flavivirus genomics

#### 2.3.1 Sequencing methods

Most complete flaviviral sequences characterized to date have been produced using complementary DNA clone(s) of the viral genome, or, more recently, following overlapping PCR amplifications along the viral genome. The latter method was optimized within the framework of the VIZIER Project: the LoPPS method, a shotgun-based approach applied to long PCR amplification products, was proven to be cost-effective and enabled the complete sequencing of large PCR products in a high-throughput format (Emonet et al., 2006, Emonet et al., 2007). More recently, high-throughput pyrosequencing methods (Margulies et al., 2005) have shown potential for the rapid characterization of viruses produced in cell cultures.

#### 2.3.2 Sequencing of previously discovered flaviviral species

Since the year 2000, significant progress has been made in the field of flavivirus genomics. Billoir et al. (2000) produced the first complete sequences of NKVs (i.e. APOIV and RBV). This was followed by the characterization of other NKVs: the MODV and Montana Myotis leukoencephalitis viruses (MMLV) (Charlier et al., 2002, Leyssen et al., 2002), YOKV (Tajima et al., 2005) and EBV (Kuno and Chang, 2006). The highly atypical Tamana bat virus (TABV) was also characterized. TABV was isolated in 1973 in Trinidad from a *Pteronotus parnelii* bat (Price, 1978) and its taxonomic position remained unresolved for nearly 30 years. Genome sequencing finally revealed that the virus was clearly, but very distantly, related to other known flaviviruses (de Lamballerie et al., 2002). The evolutionary relationship of this virus (which chronically infects bats and has no known vector) with other flaviviruses remains unclear. Complete sequences were also established for a number of “classical” arboviruses within the genus: St. Louis encephalitis virus (SLEV) (Billoir et al., 2000), Usutu virus (USUV) (Bakonyi et al., 2004), Iguape (IGUV), Bussuquara (BSQV), Kokobera (KOKV) and Ilheus (ILHV) viruses (Kuno and Chang, 2005), Alfuy virus (ALFV) (May et al., 2006), Sepik virus (SEPV) (Kuno and Chang, 2006), Kedougou (KEDV), Zika (ZIKV) and Bagaza (BAGV) viruses (Kuno and Chang, 2007), and Rocio virus (ROCV) (Medeiros et al., 2007).

The VIZIER Project has enabled full-length genome characterization of all previously identified flavivirus species. The analysis of all tick-borne flavivirus species (Grard et al., 2007) led to significant development of the previously recognized taxonomic classification, e.g. the creation of the Kadam TBV group, and of the Karshi virus species, and the assignment of TBEV and LIV to a unique species (TBEV) which included the four viral types: Western TBEV, Eastern TBEV, Turkish sheep TBEV and LIV.

Within VIZIER, similar studies devoted to other flavivirus groups have been conducted. In the Aedes-borne virus group, the complete coding sequences of Potiskum (POTV), Saboya (SABV), Jugra (JUGV), Banzi (BANV), Uganda S (UGSV), Bouboui (BOUV), Edge Hill (EHV), Sepik (SEPV), Wesselsbron (WESSV), Kedougou (KEDV), Zika (ZIKV) and Spondweni (SPOV) viruses have now been established or verified (Grard et al., in press). In the group of Culex-borne viruses (Moureau et al., unpublished data), the complete coding sequences of Aroa (AROAV), Stratford (STRV), Naranjal (NJLV), Israel Turkey (ITV), Ntaya (NTAV), Sitiawan (SV), Tembuzu (TMUV), Cacipacore (CPCV), Koutango (KOUV) and Yaounde (YAOV) viruses have been characterized. In the case of the NKV flaviviruses (Moureau et al., unpublished data), the sequences of Sokuluk (SOKV), Bukalasa bat (BBV), Dakar bat (DAKV), Batu cave (BCV), Phnom Penh bat (PPBV), Carey Island (CIV), Cowbone Ridge (CRV) and Sal Vieja virus (SVV) were obtained. In all cases, the additional information has enabled new, further analyses of a large panel of flaviviral species to be performed and provided relevant information regarding taxonomic classification and evolutionary relationships.

#### 2.3.3 Newly discovered flaviviruses

In recent years, a number of interesting atypical viruses related to known flaviviruses have been identified:

*THE CFAV GROUP*—A second virus related to CFAV, Kamiti River virus (KRV), was isolated in 2003 from African Aedes Macintoshi mosquito (Crabtree et al., 2003, Sang et al., 2003). Subsequently, field isolates of CFAV were identified from New World Aedes and Culex mosquitoes (Cook et al., 2006). Recently, a new flavivirus associated with phlebotomines has been detected by molecular biology in Algeria (Moureau et al., 2009), and another new insect flavivirus associated with *Ochlerotatus caspius*, *Ae. vexans*, *Cx. theileri*, *Anopheles atroparvus* and *Culiseta annulata* has been detected in Spain (Aranda et al., 2008). An additional insect flavivirus associated with *Culex* spp. has been also described from Japan (Hoshino et al., 2007), in Guatemala (Morales-Betoulle et al., 2008), in Mexico (Farfan-Ale et al., 2009), and in both the USA and Trinidad (Kim et al., 2009). Taken together, these studies have revealed that the genetic and ecological diversity of CFAV-related viruses is much higher than previously thought. Indeed, apparently such viruses commonly infect a large range of mosquito species all over the world and are hypothesized to be more accurately described as “insectiviruses” (as opposed to arboviruses). The discovery of long CFAV-related sequences inserted into the cellular genomes of *Aedes albopictus* and *Ae. aegypti* mosquitoes (Crochu et al., 2004) provided an unexpected and intriguing suggestion of an intimate and complex relationship between *Aedes* spp. mosquitoes and CFAV-related viruses.
*NGOYE VIRUS*—Another unique virus, “Ngoye virus” (NGOV), was identified by molecular methods from Rhipicephalus ticks sampled from Bovidae in Senegal. This virus has not yet been successfully propagated in cell cultures or newborn mice (Grard et al., 2006). It is more closely related to “classical” flaviviruses than it is to TABV, but it also constitutes a new independent evolutionary lineage within the genus Flavivirus.
*NEW AEDES ASSOCIATED VIRUSES*—Recently, Nounané virus (NOUV) was isolated from Uranotaenia in Côte d’Ivoire (Junglen et al., 2009) and Barkedji virus in Senegal (Dupressoir et al., unpublished data, GB EU078325, 2008). These viruses seem to represent a new and distinct group inside the MBV group (more information on Flavivirus phylogeny is reported as Supplementary Information).

## 3 Structure and function of flaviviral enzymes

### 3.1 The flaviviral NS3 protein

The bipartite NS3Pro-NS3Hel is an enzyme central to flavivirus replication and polyprotein processing. Dissecting the structural and functional properties of this protein in its full-length state is therefore key to improving our understanding of the flavivirus life cycle and informing the design of effective antiviral drugs. It remains unclear why NS3 harbours several catalytic activities within one polypeptide chain, however the conservation of this arrangement across the Flaviviridae genus suggests some functional relevance. Crucially, it is a matter of debate whether there is an interplay between the catalytic activities of the individual domains and whether there is a functional role for the linker region, a poorly conserved, acidic stretch of residues connecting the two domains (see below).

#### 3.1.1 NS3 protease domain

##### 3.1.1.1 Functional aspects of the NS3 protease

The 375-kDa flaviviral polyprotein precursor is processed by host proteases and a virus-encoded protease activity localized at the N-terminal domain of NS3. Whereas the cleavage at the junctions C-prM, prM-E, E-NS1, NS4A-NS4B (Speight et al., 1988, Nowak et al., 1989), and likely also NS1-NS2A (Falgout and Markoff, 1995), is performed by the host signal peptidase located within the lumen of the ER, the remaining peptide bonds between NS2A-NS2B, NS2B-NS3, NS3-NS4A and NS4B-NS5 are cleaved by the virus encoded NS3Pro. Cleavage at the NS2B/NS3 site is performed in cis, but is not necessary for protease activity (Leung et al., 2001, Bera et al., 2007). Thus, the protease activity of NS3 is essential for viral replication and its inhibition has to be considered as a valuable intervention strategy for the treatment of flaviviral infections.

**Fig. 1:**
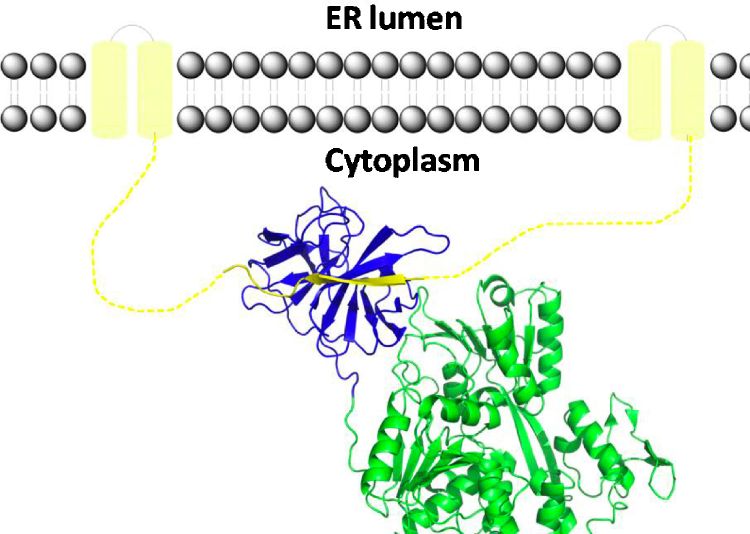
Model representation of NS3 (full-length) anchoring via NS2B to the ER membrane. The N-terminal NS3Pro domain is shown in blue, the NS3Hel domain in green. The crystal structure of DENV4 NS2B/NS3 (PDB entry 2VBC, Luo et al., 2008a) was used for model preparation. The NS2B protein is shown in yellow, modeled regions are shown as dashed lines and helices anchoring the complex to the membrane.

The activity of NS3Pro is strongly dependent on the association of a 40-amino acid region of NS2B acting as a cofactor for NS3Pro resulting in the formation of a heterodimeric complex. NS2B is a small protein (∼14 kDa) with a central hydrophilic part (residues 49–89) involved in binding to NS3, thereby fulfilling a chaperone-like role in stabilizing the latter protein, and two terminal hydrophobic regions responsible for membrane association of the NS2B/NS3 complex (Fig. 1) (Chambers et al., 1991, Chambers et al., 1993, Falgout et al., 1991, Falgout et al., 1993, Lindenbach and Rice, 2003, Lescar et al., 2008). The co-localization of NS2B and NS3 in convoluted membranes suggests these as the location for polyprotein processing by NS2B/NS3Pro, whereas Golgi-derived vesicle packets (the compartment presumably involved in RNA replication by NS3 and NS5) lack the presence of NS2B. Accordingly, the relevance of NS2B for non-proteolytic NS3 activities, such as helicase, nucleoside triphosphatase and 5′RNA phosphatase activities located in the C-terminal two-third of NS3, is yet unclear.

Even though the minimum requirements for proteolytic activity comprise the NS3 residues 1–160 (in WNV) or 1–167 in DENV2 (Li et al., 1999, Leung et al., 2001), the maximum activity concerning WNV NS3Pro, for an optimized fusion construct containing 44 NS2B residues covalently connected via a G4SG3 linker to the NS3Pro domain, has been observed for the N-terminal 1–184 residues (Chappell et al., 2007). Interestingly, a comparative analysis of the proteolytic activity of the full-length NS3 protein (1–618) fused to the optimized NS2B-G4SG3-linker region showed only marginal influence of the larger C-terminal domain on the NS3Pro kinetic parameters (Chappell et al., 2007). In contrast, studies of WNV full-length NS2B/NS3 and full-length NS3 exhibited different catalytic activities with respect to the unwinding of DNA and RNA: whereas full-length NS3 is capable to unwind both DNA and RNA templates, full-length NS2B/NS3 unwinds only RNA templates, and DNA unwinding is severely repressed (Chernov et al., 2008). Accordingly, the NS2B/NS3Pro part restricts substrate specificity of the C-terminal NS3Hel domain, however, in the absence of NS2B, the NS3 protein might dissociate from membranes and interfere with host DNA after translocation into the host-cell nucleus (Chernov et al., 2008).

##### 3.1.1.2 Three-dimensional structures determined for NS3 protease

**Fig. 2:**
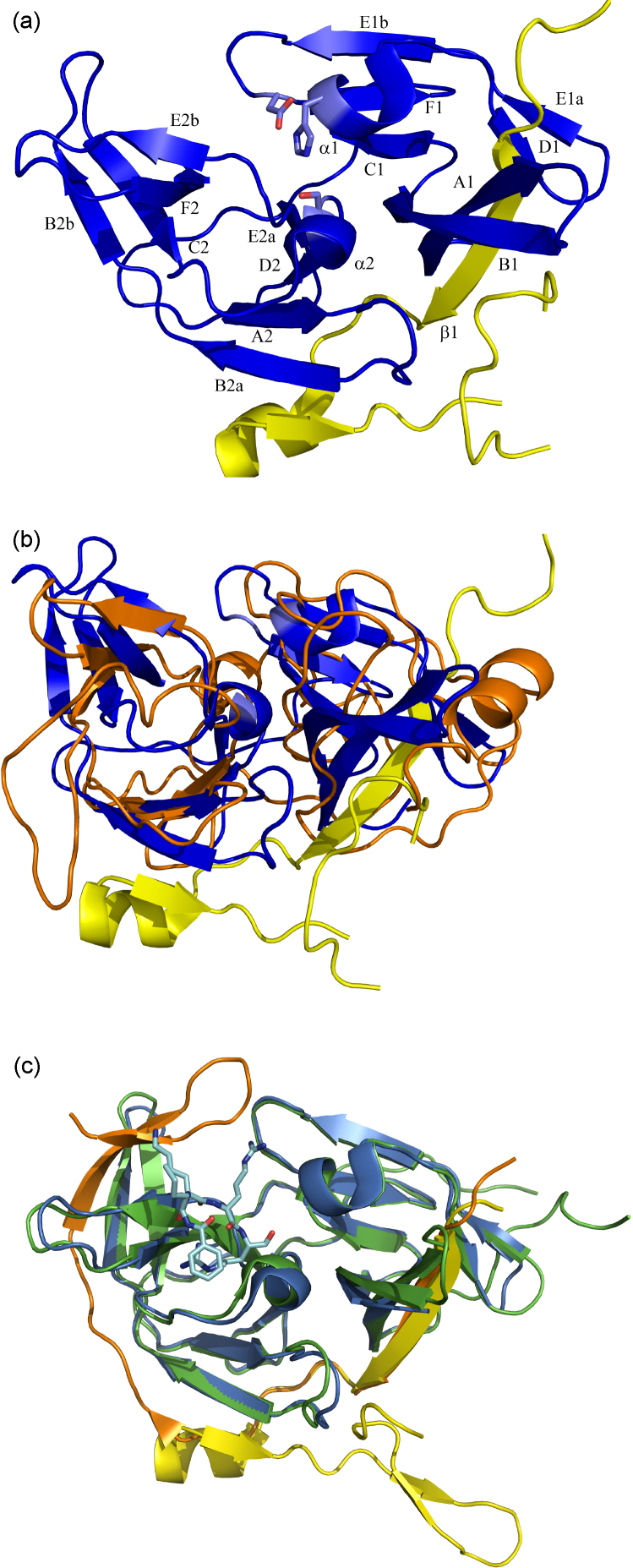
Crystal structures of NS3Pro from DENV2 and WNV viruses. (a) Overall fold of NS2B/NS3Pro from DENV2 (PDB entry 2FOM, Erbel et al., 2006). NS3Pro is shown in blue, the NS2B region, ordered in the crystal structure, is shown in yellow. (b) Superposition of DENV2 NS2B/NS3Pro as depicted in (a) and the crystal structure of DENV2 NS3Pro without the stabilizing cofactor shown in orange (PDB entry 1BEF, Murthy et al., 1999). Remarkably, substantial differences with respect to secondary structure elements are observed. (c) Superposition of the WNV NS2B/NS3Pro in ligand-bound and uncomplexed state. The NS3Pro covalently linked to the inhibitor (PDB entry 2FP7, Erbel et al., 2006) is shown in blue with the cofactor and ligand colored in orange and light blue, respectively. In the uncomplexed state (H51A mutant, PDB entry 2GGV, Aleshin et al., 2007) shown in green, the NS2B colored in yellow exhibits remarkable plasticity compared to the ligand-bound conformer.

In 1989, sequence and structural comparison studies of the flavi- and pestiviral genomes suggested the presence of a serine protease related to the trypsin family, comprising a His, Asp, Ser catalytic triad (Bazan and Fletterick, 1989, Gorbalenya et al., 1989a). One year later, this prediction was verified for YFV NS3 by mutagenesis and characterization of segments of the NS3 gene (Chambers et al., 1990). The first crystal structure of a flavivirus NS3Pro was described in 1999 (Murthy et al., 1999) for DENV2. This crystal structure served as a template for homology modeling studies and interpretation of biochemical data (Nall et al., 2004, Zhou et al., 2006). Table 2 provides an overview of the crystal structures from flavivirus NS3Pro currently available. The binding mode of a peptidic mung-bean Bowman-Birk inhibitor in complex with DENV2 NS3Pro has been reported subsequently (see below). Even though these structures helped to explain various biochemical observations, such as the redundant nature of interactions formed by Arg and Lys residues in the S1 substrate-recognition sub-site, they were substantially different from the more relevant picture represented by the recently described crystal structures of flaviviral NS3Pro in complex with its cofactor NS2B (Fig. 2a and b). Accordingly, recent structure determination attempts were focused on the crystallization of fusion proteins containing the hydrophilic part of NS2B and the NS3Pro domain, linked via a glycine-rich linker (Erbel et al., 2006, Aleshin et al., 2007, Robin et al., 2009). So far, only crystal structures of flavivirus NS3Pro from DENV and WNV have been described (Table 2).

**Table 2:** Overview about the currently PDB-deposited crystal structures of Flavivirus NS3 proteases (April 2009).

Viral Protease	Resolution (Å)	Ligand/inhibitor	PDB entry	Reference, year of publication
DV2 NS3pro	2.1	Uncomplexed	1BEF	Murthy et al. (1999)
DV2 NS3pro	2.1	Mung Bean Bowman-Birk inhibitor	1DF9	Murthy et al. (2000)
DV2 NS3pro	2.1	Mung Bean Bowman-Birk inhibitor	2QID	Murthy et al. (to be published)
DV4 NS2b/NS3 protease-helicase	3.15	Uncomplexed	2VBC	Luo et al., 2008a, Luo et al., 2008b
DV2 NS2B/NS3pro	1.5	Uncomplexed	2FOM	Erbel et al. (2006)
WNV NS2B/NS3pro	1.68	Covalently bound peptide-type inhibitor	2FP7	Erbel et al. (2006)
WNV NS2B/NS3pro, His51Ala mutant	1.8	Uncomplexed	2GGV	Aleshin et al. (2007)
WNV NS2B/NS3pro	2.3	Aprotinin	2IJO	Aleshin et al. (2007)
WNV NS2B/NS3pro	2.45	Covalently bound peptide-type inhibitor	3E90	Robin et al. (2009)

In its activated state, the flavivirus NS3Pro consists of the N-terminal domain of the full-length NS3 protein and its NS2B cofactor. The hydrophilic region of NS2B strongly interacts with NS3Pro, whereas both N- and C-terminal moieties of NS2B form two hydrophobic helices putatively acting as membrane anchors (Fig. 1). NS2B/NS3Pro adopts a chymotrypsin-like fold comprising two b-barrels, each formed by six β-strands, embedding the protease catalytic triad (His51, Asp75, Ser135) in the cleft between the two β-barrels (Fig. 2a). The presence or absence of the NS2B cofactor substantially influences the NS3Pro structure with respect to the extension and location of secondary structure elements (Erbel et al., 2006). Notably, in the cofactor-free DENV2 NS3Pro structure, the secondary structure elements are either shorter or even absent relative to DENV NS2B/NS3Pro. In the latter protein, the hydrophilic region of NS2B forms a link between the two b-barrels and contributes an anti-parallel β-strand to each of the b-barrels. The arrangement of the catalytic triad of the NS2B-bound NS3Pro suggests an exhaustive H-bonded network between the catalytic residues, in particular, a single-donor–double-acceptor (three-center) hydrogen bond between His51 and Asp75, whereas the structures lacking NS2B in the free or inhibitor-bound state exhibit an interaction geometry where only one weak H-bond between His51 and Asp75 is observed (Murthy et al., 1999, Murthy et al., 2000). These structural differences and the less constrained framework in absence of NS2B will presumably be related to the low proteolytic activity described for the non-cofactor-bound NS3Pro (Falgout et al., 1991, Falgout et al., 1993). While cleavage of small substrates by DENV2 NS3Pro is virtually not affected by the presence or absence of NS2B, degradation of larger substrates is strongly stimulated by presence of NS2B (Yusof et al., 2000).

Sharing a sequence identity of 50%, the overall fold observed for the NS2B/NS3Pro from WNV and DENV2 is very similar, with only subtle deviations in length and location of secondary structure elements (Aleshin et al., 2007). Interestingly, WNV NS2B/NS3Pro crystal structures presently available suggest conformational plasticity of the NS2B peptide: whereas in those protease structures hosting a small-molecule inhibitor in the active site, NS2B forms a belt around NS3Pro by contributing one β-strand to the N-terminal and two β-strands as β-hairpin motif to the C-terminal b-barrels, in the unbound state, the latter β-hairpin does not contribute to the C-terminal β-barrel (Fig. 2c). Instead, while the N-terminal NS2B fragment (residues 52-58) remains associated with the N-terminal b-barrel, the C-terminal residues form a short helical segment and a short β-strand interacting with strand B2a of NS3Pro, but the following hairpin motif points into the solvent and interacts with symmetry-related NS3Pro molecules. A similar fold for NS2B is observed in the inhibitor-free DENV2 NS2B/NS3Pro, with a disordered region corresponding to the b-hairpin in WNV NS2B/NS3Pro. The reasons for this unexpected NS2B plasticity are not completely understood (Erbel et al., 2006, Aleshin et al., 2007, Chappell et al., 2008). Nevertheless, the fold adopted by NS2B appears relevant for structure-based ligand design of inhibitors of WNV NS3Pro, as the NS2B b-hairpin tip in complexed NS2B/NS3Pro partly contributes to the formation of the S2 as well as the S3 pockets and may thereby directly interact with the bound ligand (see below).

##### 3.1.1.3 Flavivirus NS3 protease complexes with inhibitors

**Fig. 3:**
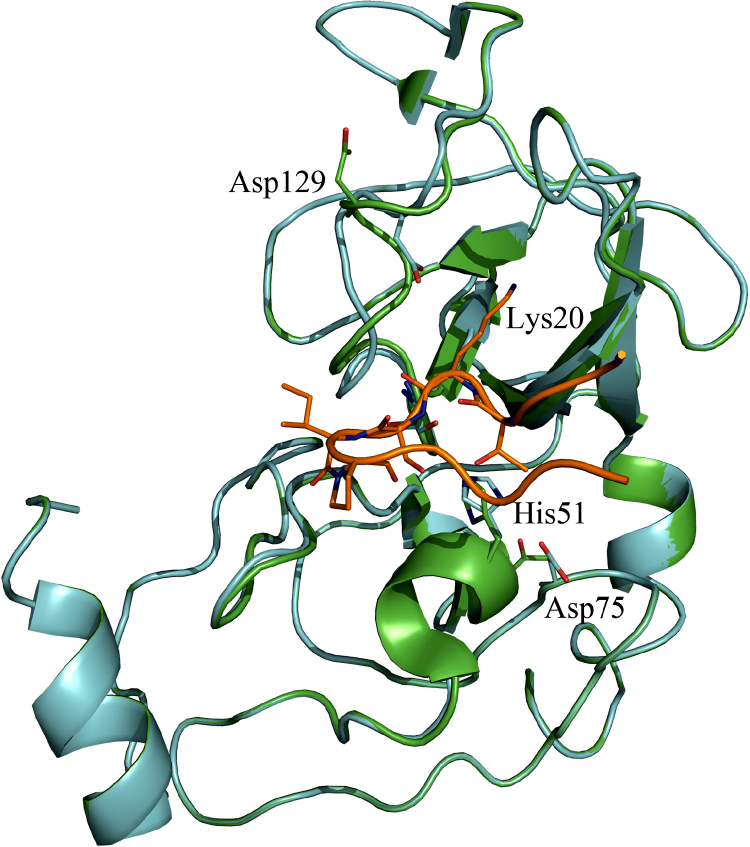
DENV2 NS3Pro complexed with a Bowman-Birk inhibitor from Mung Bean (PDB entry 1DF9, Murthy et al., 2000). The representation shows a superimposition of the two protein molecules present in the asymmetric unit and the relevant peptide region of the inhibitor (lysine head, depicted in orange). The crystal structure suggests a pronounced mobility for the region 126–136 lining the specificity pockets of the NS3Pro. Particularly Asp129 (equivalent to Asp189 in trypsin) is capable of pointing either towards the solvent or contributing to the S1 pocket.

In order to analyze the substrate preference of the proteases and to establish the basis for structure-based drug lead design, various contributions analyzed the interaction of peptide-like ligands with the protein active site (see Table 2). The first complex structure of NS3Pro lacking the NS2B cofactor described by Murthy et al. (2000) revealed the interaction of the DENV2 NS3Pro binding pocket with a polypeptide-type Bowman-Birk inhibitor isolated from mung beans, this being the only structure of a complex of DENV2 NS3 available to date. Despite the absence of NS2B, the structure allows general conclusions about the properties and ligand preference of the NS3Pro substrate-recognition pockets. The bivalent inhibitor possesses a lysine-head and an arginine-head, both occupying the substrate binding pockets of two different NS3Pro molecules simultaneously (Murthy et al., 2000). Both basic residues occupy the S1 pocket while establishing different interactions. The NS3Pro molecule hosting the inhibitor lysine head adopts virtual identical side-chain conformations as observed in the inhibitor-free NS3Pro. However, the second NS3Pro molecule exhibits strong conformational changes, particularly in the region Val126-Gly136, to adopt a binding-competent conformation (Fig. 3). The complex structure shows that Asp129 points either to the solvent (in the P1-Lys-bound molecule), or interacts with the basic residue (with P1-Arg bound) of the ligand, but the latter is involved in further charge-assisted hydrogen-bonds in a fashion obviously compensating the mutational loss of the interaction to Asp129 (Murthy et al., 2000). Additionally, a comparison of both NS3Pro molecules of the DENV2 NS3Pro-inhibitor complex reveals remarkable plasticity of active site residues (Fig. 3).

**Fig. 4:**
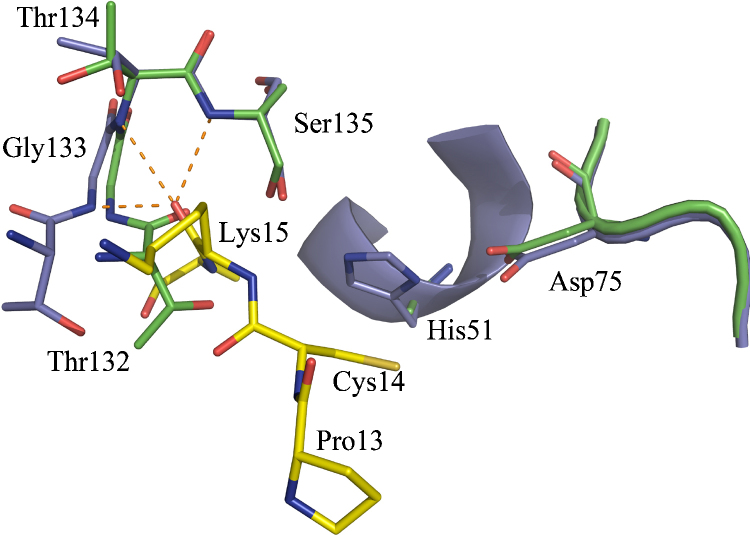
Induction of the oxyanion hole in WNV NS3B/NS3Pro by the polypeptide-type inhibitor aprotinin (for clarity, only Pro13-Lys15 are shown in yellow). Residues of the uncomplexed NS3Pro (H51A mutant, PDB entry 2GGV, Aleshin et al., 2007) are shown as green sticks, residues of the aprotinin-bound enzyme are depicted in blue (PDB entry 2IJO, Aleshin et al., 2007). The peptide bond Thr132-Gly133 flips and contributes via its backbone nitrogen atom to the formation of the oxyanion hole. H-bonding interactions between the ligand carbonyl oxygen and the backbone nitrogens of Gly133, Thr134 and Ser135 are shown as orange dashes.

The flexible behavior of DENV2 NS3Pro is not observed in the three available WNV NS3Pro-ligand complexes, all containing the NS2B cofactor. One of them hosts aprotinin as the inhibitory ligand, the other two are complexed with a peptide-type substrate analogue covalently bound to the catalytic triad residue Ser135. Whereas the overall structure of the three protein/inhibitor complexes is very similar, in contrast to the peptidic inhibitor described by Erbel et al. (2006), aprotinin occupies all the major specificity pockets of the NS3Pro (S2-S2′). Additionally, it induces a catalytically competent conformation with a fully structured oxyanion hole established by the main-chain nitrogens of Gly133 and Ser135 (Aleshin et al., 2007). In the ligand-free state, the peptide bond between Thr132 and Gly133 is flipped, thereby forming a helical 310 conformation for residues 131–135. A superposition of the two conformations observed for the aprotinin-bound and ligand-free states is shown in Fig. 4. The main chain of aprotinin residues 13–19 forms antiparallel β-sheet interactions with the strands E2B and B1 of WNV NS2B-NS3Pro. In contrast, the substrate analogue inhibitor described by Erbel et al. adopts a loop conformation supported by favourable cation–p interactions between the P1-Arg residue and the inhibitor benzoyl cap (Fig. 2c). The protein–ligand complexes provide structural evidence for the strong preference for ligands (substrate or inhibitor) comprising basic residues at the P1 and P2 sites. Next to other interactions, the properties of the S1 pocket are governed by the salt-bridge between Asp129 and P1-Arg, as well as by Tyr161 contributing a face-to-face π-cation stacking with the P1 residue. Interactions with the P2 moiety are mainly contributed by the tip of the b-hairpin formed by the NS2B cofactor whose backbone oxygen atoms of Asp82 and Gly83 and the Od1 atom of Asn84 act as acceptors of charge-assisted H-bonds donated by the hosted P2-Arg residue. The latter forms an additional H-bond to Asn152 Oδ1. A P2-Lys moiety is capable of mimicking two of these interactions (H-bonds accepted by the backbone oxygen of Gly83 and by Asn152 Oδ1), and additionally establishes a hydrogen-bond to one of the carboxylate oxygens of Asp75. However, replacement of the P2-basic residue by alanine leads to a total loss of binding (Erbel et al., 2006). The S1′ pocket is lined on one side by the catalytic His51 as well as by Gly37, providing only sufficient space to accommodate small P1′ side chains such as Gly, Ser, or Thr (Aleshin et al., 2007). The different properties and plasticity behavior of the DENV2 and WNV specificity pockets could be exploited to design substrates with selectivity for only one of the flavivirus NS3Pro. Whereas probing the cleavage activity revealed a strict substrate specificity of WNV NS2B-NS3Pro, in agreement with the described mobility of the Val126-Gly136 segment (Fig. 3), the DENV2 NS2B-NS3Pro was less selective and tolerated well the presence of a number of amino acid types at either the P1′ or the P2′ site (Shiryaev et al., 2007).

Very recently, Robin et al. (2009) described a crystal structure of WNV NS2B-NS3Pro in complex with a substrate-based tripeptide inhibitor capped at its N-terminus by a naphthoyl moiety and at its C-terminal end by an aldehyde. The latter acts as an electrophilic warhead for covalent inhibition. Interestingly, in one of the two NS3Pro molecules present in the asymmetric unit, the catalytic His51 side chain adopts a split conformation. One conformer hydrogen-bonds to the aldehyde oxygen directing it for a nucleophilic attack by the catalytic Ser from the re side, whereas the other His conformer, inconsistent with a catalytic triad, points away from the reaction center enabling the oxyanion hole to direct the nucleophilic attack from the side. These observations suggested a role for the ligand to stabilize the His in its catalytically competent conformation.

Proteases related to the occurrence of pathobiochemical processes have raised the interest of biochemists and drug designers for many years (Mittl and Grutter, 2006). Benefiting from the knowledge thereby generated, current efforts to develop flavivirus NS3Pro inhibitors suitable for clinical use are indeed promising. Due to the increasing prevalence of infections caused by pathogenic flaviviruses such as WNV, different types of DENV, and SLEV, development of anti-flaviviral drugs is of utmost importance (Ghosh and Basu, 2008). Even though lessons from the treatment of Human Immunodeficiency Virus (HIV) and HCV infections show in a dramatic way the development of escape mutations conferring resistance to viral proteases upon single therapy with only one inhibitor (Manns et al., 2007), the protease inhibitors developed do contribute to an efficient combination therapy. Since NS2B-NS3Pro is obviously not only responsible for processing of the viral polyprotein, but also appears to contribute to further pathogenic processes such as induction of membraneous structures, neurovirulence and cleavage of host cell proteins (see above), inhibition of the proteolytic activity is a promising antiviral strategy.

#### 3.1.2 NS3 helicase domain

##### 3.1.2.1 Functional aspects of the NS3 helicase domain

It is well understood that RNA synthesis by the viral replication machinery requires unwinding of the RNA secondary structure in the template RNAs. The NS3Hel domain is held to assist in initiation of (−)ssRNA synthesis by unwinding the RNA secondary structure in the 3′ UTR (Takegami et al., 1995). The key role of helicase activity in viral replication has been demonstrated through site-directed mutagenesis (Grassmann et al., 1999, Matusan et al., 2001). Crystal structures of the flavivirus C-terminal NS3RTPase/Hel domain have been solved for YFV (Wu et al., 2005), DENV (Xu et al., 2005, Luo et al., 2008b) and JEV (Yamashita et al., 2008). In the context of the VIZIER Project, three new structures have been obtained for MVEV (Mancini et al., 2007); 1.9 Å resolution), KUNV, an Australian variant of WNV (Mastrangelo et al., 2007b; 3.1 Å) and KOKV (Speroni et al., 2008; 2.1 Å). In particular, the KOKV NS3Hel domain features high thermostability and good propensity to crystallize, making this an attractive model system for structural and biochemical analysis of inhibitor binding.

##### 3.1.2.2 Three-dimensional structures determined for the NS3 helicase domain

**Fig. 5:**
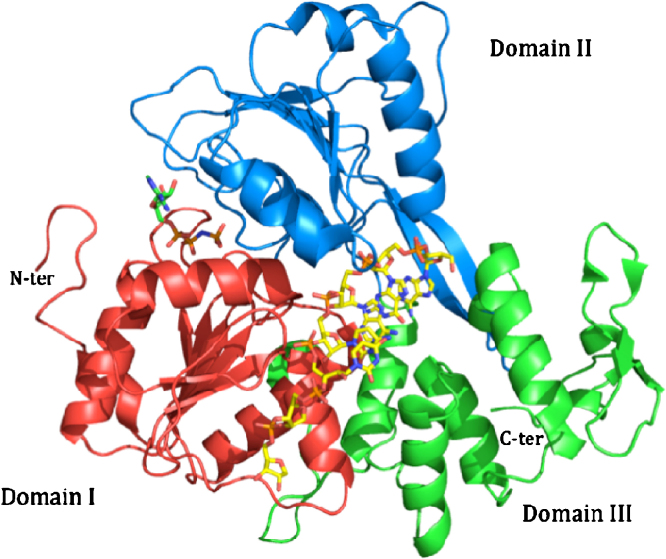
The structure of DENV NS3Hel with its three domains (I red, II blue and III green) bound to AMPPNP (left, molecule in green) and RNA (7 bases are visible: AGACUAA in yellow), adapted from PDB entry 2JLV (Luo et al., 2008a).

The flaviviral NS3Hel tertiary structure is characterized by three domains, each of about 130–150 amino acids (Fig. 5). The first two domains (domains I and II) are structurally similar, displaying an α/β open sheet topology (Rossman fold), composed of six β-strands (topology β1–β6–β5–β2–β4–β3), surrounded by four and three α-helices, respectively. Domain III is mainly composed of five approximately parallel α-helices and two antiparallel β-strands (Fig. 5). In domain II, a β-hairpin (residues 426–450) protrudes from the core domain projecting towards domain III and is held to be a critical element in unwinding activity (Luo et al., 2008b). The first two domains, likely originated by gene duplication (Caruthers and McKay, 2002), host seven characteristic sequence motifs of the NS3Hel superfamily 2 (Gorbalenya et al., 1989b, Cordin et al., 2006), associated with nucleic-acid binding and NTP hydrolysis (Caruthers and McKay, 2002). In particular, the conserved motifs I (GAGKTRR) and II (DEAH), also known as Walker A (ATP phosphate-binding loop, or ‘P-loop’) and Walker B motifs (Mg^2+^-binding; Walker et al., 1982), are located in the amino-terminal region (domain I) where they interact with the NTP substrate and Mg^2+^, respectively (Xu et al., 2005). In addition, two conserved Arg residues in domain II (Arg458 and Arg461: “arginine fingers” in motif VI) are involved in ATPase and RTPase activities (Sampath et al., 2006), and in the structural rearrangement that results in RNA unwinding, following ATP hydrolysis. A similar role in coupling the ATPase and RNA unwinding activities is played by the residues of the Ia motif, as gathered from mutagenesis studies on the KOKV NS3Hel (Speroni et al., 2008). The central region of the NS3Hel, where the three domains contact each other, hosts a cleft held to be involved in ssRNA binding during the helicase activity (Xu et al., 2005, Luo et al., 2008b).

Differences between the YFV, DENV, KUNV, KOKV, JEV and MVEV structures were found to be confined primarily to the relative orientation/distance of domain II to domains I and III, suggesting that movement of domain II can affect nucleic-acid translocation in an ATP-dependent mode according to the ‘inchworm’ model (Mancini et al., 2004, Mastrangelo et al., 2007b). Such overall structural rearrangement was recently confirmed by a detailed structural study on DENV4 NS3Hel, which describes the structures of various complexes with ATP analogues and ssRNA of 12–13 nucleotides (Luo et al., 2008b) (Fig. 5). In particular, upon ssRNA binding domain III rotates about 11° away from domain I with the simultaneous narrowing the of cleft between domains I and II (12° rotation). The overall movement can be described as an opening of both the ssRNA access site, located between α-helices α2 (domain II) and α9 (domain III; as showed in normal mode analysis; Mastrangelo et al., 2007b), and the ssRNA exit site, by the repositioning of a loop (disordered in many crystal structures) located between strands β3 and β4 in domain I.

##### 3.1.2.3 Characterization of helicase activity

RNA unwinding activities are assessed using a partially dsRNA molecule consisting of a 14 base 3′ single-stranded tail followed by a 16 base-pair dsRNA region (Wu et al., 2005). Generally, NS3Hel containing longer linker regions show higher activity than those with short linkers. The DENV4 NS3Hel (NS3 178-end) used by Luo et al. (2008b) was truncated close to boundaries shown previously for DENV, WNV and YFV to yield inactive or significantly impaired domains with respect to ATPase and helicase activities when compared to constructs with N-terminally extended linker regions (Li et al., 1999, Wu et al., 2005, Xu et al., 2005). In contrast, the MVEV NS3Hel construct includes a significantly longer linker suggesting that the observed reduction in activity for the DENV4 NS3Hel domain (Luo et al., 2008b) was due to the short linker. The reason why truncation of the linker region can have a detrimental effect on the activity of the C-terminal domain of NS3 remains unclear; it may have a functional role (Matusan et al., 2001), or cause structural artefacts as observed for KUNV NS3Hel (aa186-619) which forms a dimer (Mastrangelo et al., 2007b).

The VIZIER Project has substantially enhanced our knowledge on flaviviral NS3Hel, providing the bases for the structure-based design and development of specific antiviral molecules targeting this essential class of enzymes. The remarkable similarities in the Hel/ATPase catalytic regions indicate that it might be possible to develop compounds with a broad spectrum of activities – i.e. which are able to act on different flaviviral enzymes – and/or lead molecules that can be targeted to a specific viral enzyme through minimal ad hoc chemical modifications. Medicinal chemistry studies on protein kinases have shown that the most effective inhibitors are conformationally based; they exert their inhibitory effect through an allosteric alteration of the equilibrium among different protein conformations (Vajpai et al., 2008). Likewise, future drug-design studies on flaviviral NS3Hel will benefit from our improved understanding of the role of the various fingerprint residues and of the conformational changes that underlie the coupling between ATP hydrolysis and RNA unwinding activity.

#### 3.1.3 The full-length NS3 protein

##### 3.1.3.1 Functional aspects of the NS3 protein

Understanding the biologically relevant functional properties of NS3 is complicated by the fact that in infected cells NS3 acts anchored to or in close proximity of membranes (Lindenbach and Rice, 2003), whereas most *in vitro* characterization has been done in solution. Furthermore, as the virus progresses towards maturation, different protein–protein and protein–RNA interactions occur which demarcate specific points in its life cycle, although the details remain unclear. Polyprotein processing and replication occur in distinct, membrane-bound compartments (convoluted membranes and vesicle packets, respectively), and in each compartment, NS3 engages with different proteins (Mackenzie, 2005). An intriguing finding has been the apparent absence of NS2B, the essential cofactor of the NS3Pro, in vesicle packets (Westaway et al., 1997, Mackenzie, 2005) suggesting that in the transition from polyprotein processing to replication, the NS3Pro becomes inactive. Finally, the structure and dynamics of the polyprotein as it emerges following translation remain largely unexplored and little information exists on interactions between NS3 and other parts of the polyprotein, which might be important for priming NS3Pro for its first and subsequent cleavage activities.

##### 3.1.3.2 Three-dimensional structures determined for the NS3 protein

**Fig. 6:**
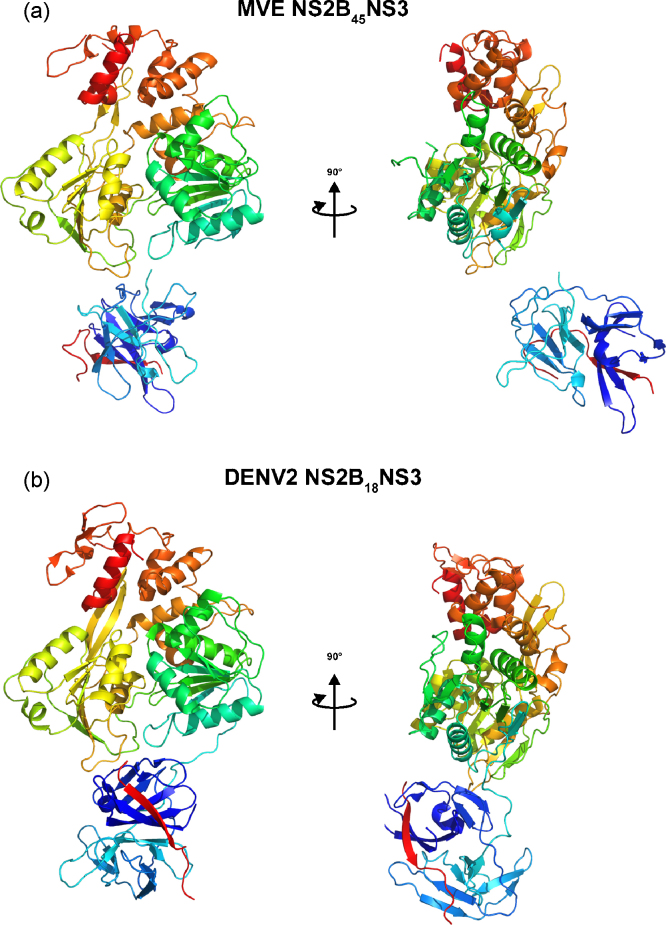
Comparison of the NS2B-NS3 structures of MVEV (upper panel) and DENV4 (lower panel); in both panels the NS3Hel domain is in the upper part of the figure, the NS3Pro domain is in blue–cyan colors, hosting the NS2B segment (red color).

Two full-length NS3 structures have been solved by X-ray crystallography: those of DENV4 NS3 (Luo et al., 2008a) and of MVEV NS3 (Assenberg et al., submitted for publication) (Fig. 6). Both structures were solved in the presence of a fragment of protein NS2B, the essential co-factor and activator for NS3Pro, by producing a single polypeptide chain where this region was linked via a flexible tether to NS3. One difference between the two studies is that for DENV4 only part of the NS2B activating region was coupled to NS3 (18 amino acids of NS2B, DENV4 NS2B_18_NS3), whereas for MVEV the full activating region was included (45 residues of NS2B, MVEV NS2B_45_NS3).

In both the DENV4 NS3 (Luo et al., 2008a) and MVEV NS3 (Assenberg et al, submitted for publication) structures the NS2B-NS3 molecule consists of two separate globular folds linked by a short linker, an arrangement consistent with SAXS data for full-length KUNV NS3 in solution (Mastrangelo et al., 2007b). The individual domains are very similar between the two molecules (r.m.s.d. of 1.5 and 1.6 Å for the NS3Hel and NS3Pro, respectively) and to the structures of isolated domains. Yet the relative orientations of the NS3Pro and NS3Hel domains are dramatically different between MVEV NS2B_45_NS3 and DENV4 NS2B_18_NS3. When superimposing the NS3Hel domains of the two structures, a rotation of ∼180° and translation of 17 Å are required to align the NS3Pro domains. The 13-residue “linker” between the NS3Pro and NS3Hel domains (residues 169–181) is ordered in DENV4 NS2B_18_NS3 but partially disordered in MVEV NS2B_45_NS3. Even though the buried surface area between the NS3Pro and the NS3Hel in DENV2 NS2B_18_NS3 is only 568 Å^2^, the NS3Pro domain and the linker loop engage in possibly significant interactions with subdomains 1 and 2 of NS3Hel. Specifically, the linker interacts with the catalytic P-loop of the NS3Hel, which assumes the distinctive apo conformation seen in the NS3Hel domain crystal structure in the absence of bound nucleotides. These interactions are not seen in the MVEV NS2B_45_NS3 structure where the buried surface area is only 30 Å^2^; however, the P-loop is found in the same apo conformation. Thus, a possible role for the linker loop could be to stabilize the apo conformation of the P-loop, in line with recent studies suggesting a functional role for the linker (truncating this acidic linker can have a substantial effect on the activity of isolated NS3Hel domains; Li et al., 1999, Wu et al., 2005, Mastrangelo et al., 2007b).

A striking observation relating to the influence of the NS3Pro domain on the NS3Hel domain emerges from a comparison of the two structures. In DENV4 NS2B_18_NS3, the interactions between NS3Pro and NS3Hel are such that motions of domain 2 of the NS3Hel, known to be important for helicase activity, would be constrained. In the MVEV NS2B_45_NS3 structure in contrast, such interactions are weak and therefore there are no constraints on the motility of the NS3Hel domain. This would suggest that the NS3Pro domain might repress helicase activity and that such activity might be regulated by switching between various transient configurations, such as those observed in the two structures. This conclusion contrasts that of Luo et al. (2008a), who saw an increased affinity for ATP when the activity of the DENV4 NS2B_18_NS3 protein was compared to that of the isolated NS3Hel domain. Although this enhancement was explained by a positive contribution of the NS3Pro domain to the electrostatic potential of the NS3Hel nucleotide binding pocket raising helicase activity, an alternative explanation is that the isolated NS3Hel domain chosen for comparison was ‘hobbled’ by truncation of the linker. Indeed the truncation used has been shown to significantly reduce helicase activity of DENV2 and other flavivirus NS3Hel domains (Li et al., 1999, Wu et al., 2005). The latter interpretation is supported by biochemical analysis of the helicase activity of MVEV NS2B_45_NS3, which showed no significant difference between the activity of full-length MVEV NS2B_45_NS3 and that of the isolated NS3Hel domain (using a more appropriate linker than in the DENV4 study). Finally, NS2B is not found in vesicle packets and therefore not part of the replication complex (Westaway et al., 1997, Mackenzie, 2005), posing further questions over the role of the NS3Pro domain in regulating helicase activity since in the absence of NS2B the NS3Pro domain partially unfolds.

##### 3.1.3.3 General structural properties of the NS3 protein

The structures of full-length NS3 raise two important questions: (1) do these structures represent two distinct and stable conformations of NS3 possibly adopted at different stages of the flavivirus life cycle or are they merely snap-shots of a highly dynamical interconversion process and (2) given the segregated nature of the two catalytic domains, what is the functional significance of this arrangement?

**Fig. 7:**
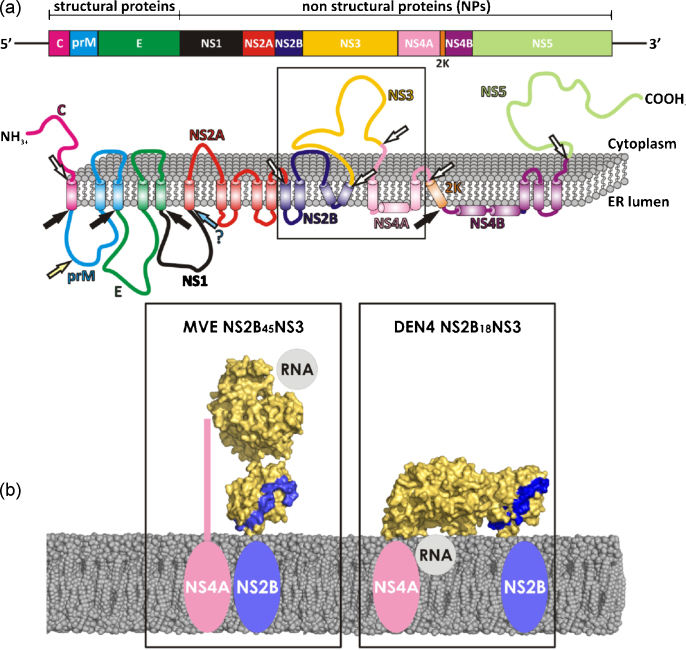
Models for membrane association of MVEV and DENV4 NS2B-NS3. (a) Schematic diagram of the flavivirus polyprotein organization and processing. The upper figure shows the linear organization of the structural and non-structural proteins within the polyprotein. The lower figure shows the putative membrane topology of the polyprotein, as predicted from biochemical and cellular analyses, which is then processed by cellular and viral proteases (denoted by arrows). (b) Predicted structural organization of MVEV NS2B_45_NS3 and DENV4 NS2B_18_NS3 at the cellular membrane. A model for the membrane is shown as van der Waals balls, atomic structures are shown in a surface representation and color coded according to the following convention: NS3 protein (pale yellow) and NS2B stretches (blue). The NS4A (shown schematically in pink) was positioned at the NS3 C-terminus (domain3) and the RNA (shown schematically in grey) is positioned in the ssRNA binding groove.

Our analysis suggests that the relative domain organization is probably highly dynamic, given the linker flexibility (disordered in the MVEV structure) and the small buried surface areas between the two domains in both structures. Further, the configurations are in principle inter convertable via simple rotations around the linker loop, and linker flexibility is probably paramount to the NS3 activities and its ability to associate with other proteins and RNA. On the other hand, specific configurations may be stabilized during a particular stage of the virus life cycle. To gain further insights into these issues, we modeled the MVEV and DENV NS2B-NS3 structures in the presence of a membrane (Assenberg et al., submitted for publication). Previous studies have shown that when in complex with NS2B, and in particular when fully activated, NS3Pro sits in a rather tight membrane-anchored sling (Clum et al., 1997, Robin et al., 2009). Fig. 7 shows a model of MVEV and DENV4 NS2B-NS3, with their NS3Pro domains superimposed and associated to the membrane as inferred from the anchoring of the published NS2B-NS3Pro structures (Erbel et al., 2006, Robin et al., 2009). In this model, the DENV4 NS2B_18_NS3 NS3Hel is positioned near the membrane with the active site orientated towards the membrane and with little space to accommodate RNA, whilst in the MVEV structure the NS3Hel domain is positioned away from the membrane, with the active site facing the cytoplasm. Thus, the DENV4 NS2B-NS3 structure appears incompatible with RTPase/helicase activity. Although this could be taken as an argument that the DENV4 conformation may not be physiologically relevant, the strength of conformational constraints imposed by the cellular environment is difficult to assess. Prior to cleavage of the NS3-NS4A junction, NS3 is also anchored to the membrane at its C-terminus via the membrane-bound NS4A. However, although there are 50 residues separating the NS3 C-terminus and the first trans-membrane α-helix of NS4A (Miller et al., 2007), the structure of the first 50 residues of NS4A remains unknown. Thus, the presence of NS4A may limit the ability of NS3 to change its conformation *in vivo*. This leads to the interesting possibility that NS3 may adopt this conformation during polyprotein processing where helicase activity is probably not wanted. Formation of the replication complex, where the helicase activity is presumably needed, would release NS2B, inactivating the NS3Pro domain. In this view, the MVEV NS2B-NS3 conformation is likely to be relevant later in the virus life cycle, during the assembly and functioning of the replication complex. The regulation of the activities of NS3 by an environment-dependent re-configuration of the molecule offers a simple temporal and spatial control mechanism, coupling activities appropriately with the virus life cycle. This model provides answers to both of the questions posed in the previous paragraph.

*In vivo*, the situation is probably complicated by the modulation of the structure and function of NS3 by additional binding partners. Thus, the activity of NS3 may be affected by interactions within the polyprotein (Zhang and Padmanabhan, 1993), and NS3 binds to free NS5 (Johansson et al., 2001, Yon et al., 2005), NS4A (Mackenzie et al., 1998) and NS4B (Umareddy et al., 2006) as well as viral RNA. In particular, it has been suggested that the C-terminal domain of NS3 binds NS5 (Liu et al., 2002, Wu et al., 2005) during the formation of the replication complex. Unfortunately, the details of these interactions remain poorly understood. Clearly, further studies are required to test the functional significance of the two conformations *in vivo*, as well as the influence of the interactions between NS3 and other viral proteins, RNA, and lipids on the conformation of NS3.

The structures of full-length NS3 reveal that the molecule can assume two radically different configurations, defined by the relative positioning of the NS3Pro and NS3Hel via a flexible inter-domain linker. We suggest that these may be important in its interactions with other proteins and RNA and, possibly, in modulating the switch to helicase and triphosphatase activities during replication.

### 3.2 The flaviviral NS5 protein

With a molecular mass of about 100 kDa, NS5 is the largest flaviviral protein; NS5 is also the most conserved one across the genus. Early on a motif of AdoMet-dependent MTases was identified within the N-terminal domain of NS5 (Koonin, 1993) whereas RdRp motifs were identified in the C-terminal domain of protein NS5 (Rice et al., 1985, Poch et al., 1989, Koonin, 1991, Bruenn, 2003). The MTase functions were demonstrated in 2002 and 2007 using the recombinant N-terminal MTase domains of DENV2 and WNV (Egloff et al., 2002, Ray et al., 2006). The RdRp activity was first demonstrated by the use of NS5-specific antisera that inhibited RdRp activity in assays using DENV2-infected cell lysates (Bartholomeusz et al., 1994), as well as by DENV1 recombinant NS5 (Tan et al., 1996). The latter bound RNA template and showed RdRp activity as detected by the incorporation of radiolabel into a neosynthesized RNA strand (Tan et al., 1996). NS5 of flaviviruses has subsequently been expressed in various *in vitro* systems, and shown to have RdRp activity (Ackermann and Padmanabhan, 2001, Guyatt et al., 2001, Nomaguchi et al., 2003, Nomaguchi et al., 2004, Kim et al., 2007). It has been demonstrated that NS5 initiates RNA synthesis de novo (i.e. primer-independent) (Ackermann and Padmanabhan, 2001, Nomaguchi et al., 2004, Selisko et al., 2006).

The N-terminal boundary of the RdRp domain of protein NS5, which comprises around 900 amino acids, has long remained unknown. Usually two nuclear localization sequences (NLS) localized between amino acid residues 320 and 405 were supposed to represent the inter-domain region between MTase and RdRp (Brooks et al., 2002). Within the VIZIER Project, structure-based sequence analysis of NS5 was conducted and allowed the definition and subsequent production of a recombinant soluble and enzymatically active RdRp domain of DV2 (NS5Pol_DV_) and WNV (NS5Pol_WNV_) starting at DV2 NS5 residue 272 (Selisko et al., 2006). More recently, we expressed full-length NS5 proteins of two strains (Vasilchencko and Oshimo) of TBEV in *Escherichia coli* (cloned in pDest14 and expressed as described, Selisko et al., 2006). The recombinant proteins were purified by IMAC followed by size exclusion chromatography. NS5 was obtained but also to a large extent a degradation product of around 30 kDa. Western blot analysis against the N-terminal hexahistidine-tag revealed that this pool was the N-terminal part of NS5. The mass of these proteins were checked by matrix-assisted laser desorption ionization-time of flight mass spectrometry (MALDI-TOF) rendering a unique peak at 30,299 Da for NS5 of TBEV Vasilchenko and 30,335 Da for NS5 of TBEV Oshima (data not shown). These data suggest that the main cleavage occurs after Arg-264 or Cys-265, depending on the presence or not of the start methionine that can be, in *E. coli*, removed. In summary, we conclude that the linker region between the MTase and the RdRp domain of flavivirus NS5 can be assigned to residues 266 to 272. Interestingly, all solved NS5MTase structures (see Section 3.2.1), which were obtained either from protein constructs consisting of about 265 or of 293 residues, comprise approximately 265 residues only, since the 266–293 stretch of the long constructs are usually disordered. The only exception are two WESSV NS5MTase domain structures (PDB entries 3ELD and 3ELU, Bollati et al., 2009b), which include the linker region and a C-terminal helix from residues 274 to 285. Interestingly, residues 267–269 are disordered, what supports our proposal of the linker region. The C-terminal region is nevertheless characterized by high mobility, which may be functional for the interaction between the NS5MTase and the NS5RdRp domains of the full-length viral protein.

#### 3.2.1 NS5 methyltransferase domain

##### 3.2.1.1 Functional background aspects of the NS5 methyltransferase domain

The flavivirus RNA is decorated with a conserved type-1 cap (N7meGpppA2′Ome-RNA) at its 5′-end, a unique structure consisting of an inverted guanosine linked to the first transcribed RNA nucleotide by a 5′–5′ triphosphate bridge. Viral MTases are involved in the mRNA capping process, transferring a methyl group from the cofactor S-adenosyl-l-methionine (AdoMet) onto the N7 atom of the cap guanine and onto the 2′OH group of the ribose moiety of the first RNA nucleotide. In the genus Flavivirus, both (guanine-N7)-methyltransferase (N7MTase) and (nucleoside-2′-O-)-methyltransferase (2′OMTase) activities have been associated with the N-terminal domain of the viral NS5 protein (Egloff et al., 2002, Ray et al., 2006, Zhou et al., 2007).

##### 3.2.1.2 Three-dimensional structures determined for the NS5 methyltransferase domain

Crystal structures of the NS5MTase domain have been reported for different mosquito-borne flaviviruses, such as DENV (Egloff et al., 2002), WNV (Zhou et al., 2007) and YFV (Geiss et al., 2009). In the context of the VIZIER Project new high-resolution structures of NS5MTases have been obtained for MEAV, a TBV (Mastrangelo et al., 2007a), for the MBV MVEV (Assenberg et al., 2007) and WESSV (Bollati et al., 2009b), and for two NKVs: YOKV (Bollati et al., 2009a) and MODV (Jansson et al., 2009) (Table 3). Moreover, structures of DENV NS5MTase, MVEV NS5MTase and WESSV NS5MTase in complex with GTP or several cap analogues, GpppA/G and N7meGpppA/G (Egloff et al., 2002, Egloff et al., 2007, Assenberg et al., 2007, Bollati et al., 2009b) have been reported, shedding light on the substrate-binding mode during methylation and on the enzyme mechanism of action (Table 3).

**Table 3:** Flaviviral MTases crystal structures.

Virus	PDB ID	Ligand(s)	Reference	Vizier
WESSV	3ELY	AdoHcy	Bollati et al. (2009a)	Yes
	3ELU	AdoMet	Bollati et al. (2009a)	Yes
	3ELW	AdoMet + GpppG	Bollati et al. (2009a)	Yes
	3EMB	AdoMet + N7MeGpppG	Bollati et al. (2009a)	Yes
	3ELD	Sinefungin	Bollati et al. (2009a)	Yes
	3EMD	Sinefungin + N7MeGpppA	Bollati et al. (2009a)	Yes

MVEV	2PX2	AdoHcy	Assenberg et al. (2007)	Yes
	2PX4	AdoHcy	Assenberg et al. (2007)	Yes
	2PX5	AdoHcy	Assenberg et al. (2007)	Yes
	2PXC	AdoMet + GpppA	Assenberg et al. (2007)	Yes
	2PX8	AdoHcy + N7MeGTP	Assenberg et al. (2007)	Yes
	2PXA	AdoHcy + GpppG	Assenberg et al. (2007)	Yes

DENV	1L9K	AdoHcy	Egloff et al. (2002)	No
	3EVG	AdoHcy	Geiss et al. (2009)	No
	1L9K	AdoHcy + Ribavirin triphosphate	Egloff et al. (2002)	No
	2P41	AdoHcy + N7MeGpppG2′OMe	Egloff et al. (2007)	Yes
	2P40	AdoHcy + N7MeGpppG	Egloff et al. (2007)	Yes
	2P3Q	AdoHcy + GpppG	Egloff et al. (2007)	Yes
	2P3O	AdoHcy + N7MeGpppA	Egloff et al. (2007)	Yes
	2P3L	AdoHcy + GpppA	Egloff et al. (2007)	Yes

YFV	3EVA	AdoHcy	Geiss et al. (2009)	No
	3EVB	AdoHcy	Geiss et al. (2009)	No
	3EVC	AdoHcy + GTP	Geiss et al. (2009)	No
	3EVD	AdoHcy + GTP	Geiss et al. (2009)	No
	3EVE	AdoHcy + GpppA	Geiss et al. (2009)	No
	3EVF	AdoHcy + N7MeGpppA	Geiss et al. (2009)	No

WNV	2OY0	AdoHcy	Zhou et al. (2007)	No
MEAV	2OXT	AdoMet	Mastrangelo et al., 2007a, Mastrangelo et al., 2007b	Yes
YOKV	3GCZ	AdoMet	Bollati et al. (2009b)	Yes

**Fig. 8:**
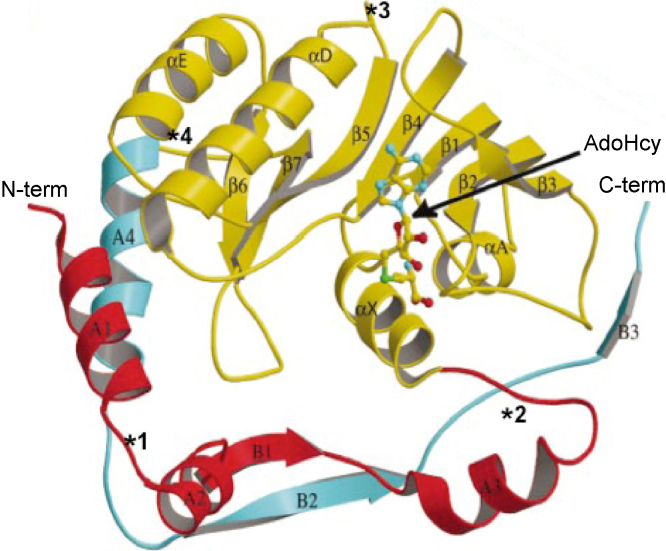
Crystal structure of DENV NS5MTase in complex with AdoHcy. A ball-and-stick representation is used for AdoHcy, whereas DENV NS5MTase is drawn as a ribbon (Egloff et al., 2002). The loops differing between NS5MTases representative of the three Flaviviral branches are highlighted with a star and an identification number referring to what has been described in the text.

The flaviviral NS5MTase domain consists of a 33-kDa protein comprising residues 1–260/270 of the N-terminus of the NS5 protein. It is characterized by an overall globular fold consisting of a core domain (residues 59–224) flanked by an N-terminal region (residues 1–58), and a C-terminal region (residues 225–265) (Fig. 8). The core domain comprises a seven-stranded β-sheet surrounded by four α-helices and two 310 helices, closely resembling the topology observed in the catalytic domain of other AdoMet-dependent MTases (Fauman et al., 1999, Bugl et al., 2000, Egloff et al., 2002). The N-terminal segment comprises a helix-turn-helix motif followed by a β-strand and an α-helix. The C-terminal region consists of an α-helix and two β-strands (Fig. 8). The core subdomain hosts the active site and the cofactor binding site (Ingrosso et al., 1989, Egloff et al., 2002, Egloff et al., 2007, Assenberg et al., 2007, Mastrangelo et al., 2007a, Zhou et al., 2007, Bollati et al., 2009a, Bollati et al., 2009b). In all the structures a cofactor molecule, in some cases the co-product AdoHcy, originated from *E. coli* and co-purified with the enzyme, is bound in this binding site, where it is stabilized by a network of hydrogen bonds and van der Waals contacts involving several residues – Ser56, Gly86, Trp87, Thr104, Leu105, His110, Asn131, Val132, Asp146, and Ile147 (residue numbering refers to WESSV NS5MTase) – and a series of interactions that are well conserved within the flaviviruses NS5MTases (Fauman et al., 1999, Egloff et al., 2002, Egloff et al., 2007, Assenberg et al., 2007, Mastrangelo et al., 2007a, Zhou et al., 2007, Bollati et al., 2009a, Bollati et al., 2009b).

The known flaviviral NS5MTases show a large degree of structural homology (r.m.s.d. < 1 Å), which reflects the high amino acid sequence conservation (between 30% and 90%). Comparison of the NS5MTases representative of each of the three flaviviruses branches (the NKV, YOKV NS5MTase; the TBFV, MEAV NS5MTase, and the MBFV, DENV NS5MTase) shows that most differences in the structures are caused by surface-loop flexibility and amino acid variability, displayed in four regions of the enzyme: (1) helix-loop-helix motif in the N-terminal domain, involved in the binding of the substrate (residues Gly21-Lys22-Thr23 in YOKV NS5MTase, substituted with Gly-Lys-Ser in DENV NS5MTase, and Thr-Lys-Glu in MEAV NS5MTase); (2) α3-αX loop (Asn47-Ile53; insertion of one amino acid in MEAV NS5MTase); (3) αD-β5 loop (Leu172-Thr178; deletion of two amino acids in DENV NS5MTase); and (4) α4-β8 loop (Leu246-Thr252; deletion of two amino acids in MEAV NS5MTase) (highlighted in Fig. 8) (Bollati et al., 2009a).

##### 3.2.1.3 Structures of protein/ligand complexes

In the context of the VIZIER Project, structures of different NS5MTases in complex with cofactor and several capped substrate analogues have been solved (Egloff et al., 2002, Egloff et al., 2007, Assenberg et al., 2007, Bollati et al., 2009b). All the complexes show that the cap guanine moiety (methylated or not) binds to a site next to the N-terminal helix-turn-helix motif, called the high affinity binding site (HBS) formed by residues Lys13, Leu16, Asn17, Leu19, Phe24, Ser150, Arg197, Arg213, Ser215. In particular, Phe24 plays an essential role in driving the cap binding by stacking against the cap guanine base during the interaction (Egloff et al., 2002, Egloff et al., 2007, Assenberg et al., 2007, Mastrangelo et al., 2007a, Bollati et al., 2009b, Milani et al., 2009). The first nucleotide after the cap is often disordered, reflecting a lack of strong interactions with the enzyme. This is in line with other studies showing that a minimal number of nucleotides in capped RNA are required for substrate binding and methylation (Ray et al., 2006, Egloff et al., 2007, Dong et al., 2008, Selisko et al., in press). A unique conformation of the cap analogue is adopted by GpppA in complex with DENV NS5MTase. The guanine is bound in the HBS and GpppA displays an overall hairpin-like shape in which the two bases stack against each other in a way that allows to accommodate a longer capped RNA because the 3′-position of the A ribose is oriented towards a positively charged surface region (Egloff et al., 2007). It was proposed that this conformation might mimic the reaction product of a putative flavivirus GTase activity: pppG + ppAGN leading to GpppAGN + pyrophosphate. Such a guanylyltransfer reaction would be the only occasion where the GTP-binding site of DENV NS5MTase needs to accommodate a non-methylated GTP.

Since flaviviral NS5MTases display both N7MTase and 2′OMTase activities (Ray et al., 2006, Egloff et al., 2007, Zhou et al., 2007, Dong et al., 2008), in order for capped RNA to be methylated at the two different sites, it must adopt two distinct binding modes relative to the enzyme active site. To date structural details on catalytically relevant states for both N7 or 2′O methyl transfer are missing. Nevertheless, it has been speculated that when the cap is bound in the HBS, the 2′OH group of the ribose moiety of the nucleotide following the cap is the closest atom to the AdoMet exchangeable methyl group (Egloff et al., 2007, Mastrangelo et al., 2007a, Bollati et al., 2009b). For this reason, the HBS is assumed to host the cap during 2′O methylation. Moreover, structural considerations suggest the existence of a secondary, putative low-affinity binding site (LBS) located in a positively charged region close to the AdoMet binding site which could be involved in binding the capped RNA substrate (Egloff et al., 2007, Mastrangelo et al., 2007a, Dong et al., 2008, Bollati et al., 2009a, Bollati et al., 2009b).

In this context, two different models of the mechanism of action of the flaviviral NS5MTase have been proposed. Model 1 (Egloff et al., 2007, Mastrangelo et al., 2007a, Zhou et al., 2007, Dong et al., 2008, Bollati et al., 2009a, Bollati et al., 2009b, Milani et al., 2009) suggests that when the capped RNA substrate binds in the LBS, it undergoes methyl transfer in position N7 of the guanine with the cap guanine fixed in the active site. Afterwards, the NS5MTase slides on the RNA chain positioning the cap in the HBS and locating the first RNA adenine in the active site for 2′O methylation. In this binding mode, the rest of the RNA chain is still stabilized by the interaction with the residues of the LBS, but shifted by one nucleotide. The analysis of conserved residues within flaviviral NS5MTases shows that there is an almost continuous and conserved region extending away from the active site towards the back of the protein—residues Tyr89, Pro113, Gly120, Trp121, Asn122, Leu123, Ile124, Phe126, Lys127, Asp131, Gly263, Thr264 and Arg265 on the side of the NS5MTase, and residues Ala60, Trp64, Leu207, Val208, Arg209, Pro211, Met220 and Arg244 on the back (numbering referring to YOKV NS5MTase (Bollati et al., 2009a). This region may play a role in stabilization of the rest of the RNA chain following the cap (no crystal structure available so far) (Bollati et al., 2009a). In order to predict the interactions between the protein and the RNA during N7 methyl transfer, Milani et al. presented a model of a short capped RNA (GpppAGUp) bound to the LBS of WESSV NS5MTase (Milani et al., 2009). The model produced after 9 ns of Molecular Dynamic simulation shows that the cap guanine was located close to AdoMet and to residues Glu149 and Arg213; other residues found to interact with capped RNA were: Arg37, Arg41, Leu44, Ser56, Arg57, Arg84, Glu149, Lys112, Ser150, Arg160, Ser215. In line with this model, it has been recently shown that point mutations of a good part of these amino acids (Arg37, Arg57, Arg84, Trp87, Glu149, Arg213, and Tyr220) dramatically inhibit N7 methyl transfer activity in WNV NS5MTase (Dong et al., 2008).

**Fig. 9:**
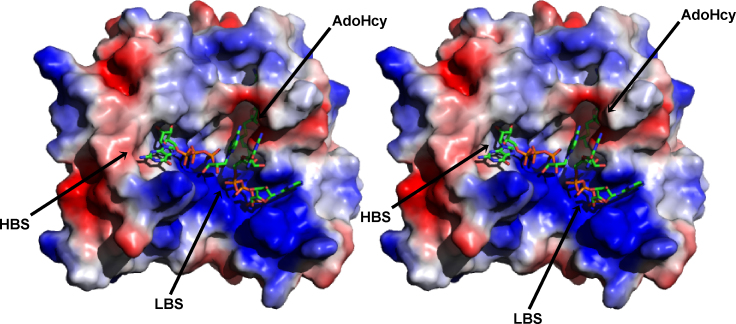
Stereo view of the complex formed by MVEV NS5MTase with AdoHcy and two molecules of GpppA: the first cap analogue binds in the HBS, the second is adjacent and interacting with the positively charged residues near the AdoMet-binding cleft in the LBS (Assenberg et al., 2007).

Based on the structure of MVEV NS5MTase in complex with GpppA, which reveals a crystallographic dimer with two GpppA molecules bound per monomer, Assenberg et al. (2007) proposed Model 2. In this structure, the first cap analogue binds in the HBS with the adenosine base approaching the AdoMet binding pocket. The second analogue binds to the LBS and stacks against the first cap analogue, with the guanosine base stacking against the adenosine base of the first analogue (Fig. 9) (Assenberg et al., 2007). Although this indicates that the LBS can bind cap analogues, supporting in this way Model 1, the authors note that in the MVEV co-crystal structure the N7 and 2′O guanosine group of the two analogue bound to the dimer are too far away (>10 and >8 Å, respectively) from the AdoMet methyl leaving group to be catalytically relevant. This casts some doubt over the catalytic relevance of the structure and indeed the significance of the LBS in controlling N7MTase activity. Further analysis of the crystallographic dimer show that two LBS regions form a nearly continuous positively charged groove, with the four GpppA molecules interacting in a manner resembling that of a continuous strand of RNA. Based on this, Model 2 proposes that the dimerization of two NS5MTase monomers generates a large positively charged RNA binding cleft that facilitates binding and translocation of the capped RNA. As the RNA moves into the catalytic site of the first monomer, N7 methylation occurs, followed by RNA translocation via the LBS to the HBS of the second monomer where 2′O methylation occurs. In this model the LBS acts merely as an RNA-binding domain, in contrast to Model 1 discussed above.

##### 3.2.1.4 Functional characterization

The first evidence of enzymatic activity from a flavivirus NS5MTase domain was demonstrated with DENV NS5MTase using short capped RNA substrates N7me ± GpppAC5 (Egloff et al., 2002). Such short substrates, even starting with GpppAG (the first two nucleotides strictly conserved in the 5′-end of the flavivirus genome), support specifically the 2′OMTase activity (Kroschewski et al., 2008, Lim et al., 2008). In the context of the VIZIER Project, a protocol was set-up to produce pure N7me ± GpppACn substrates of varying chain lengths (*n* = 1–7) in high amounts (Peyrane et al., 2007). The substrates were used to prove 2′OMTase activity in a variety of flavivirus NS5MTase domains (Mastrangelo et al., 2007a, Bollati et al., 2009a, Bollati et al., 2009b) and set-up inhibition assays (Luzhkov et al., 2007, Milani et al., 2009, Selisko et al., in press). Interestingly, it was found that DENV 2′OMTase activity and binding increases with the substrate chain length until they reach a plateau at *n* = 5. N7meGpppAC5 substrates were used to determine kinetic parameters of the 2′OMTase activity of DENV NS5MTase (Selisko et al., in press). On the other hand, in order to measure N7MTase activity of flavivirus NS5MTase domains, a substrate of 74 nucleotides is needed, which contains a conserved stem-loop structure (Ray et al., 2006, Dong et al., 2007). In the context of VIZIER, capped subgenomic RNA of DENV was produced and used to demonstrate N7MTase activity of DENV and WESSV NS5MTases (Milani et al., 2009). Moreover, in accordance with the hypothesis that the flavivirus NS5MTase domain contains the GTase activity, the WESSV NS5MTase domain was shown to covalently bind GMP at Lys28 (Bollati et al., 2009b). Following the classic scheme of cap formation (Furuichi and Shatkin, 2000) to complete the guanylyltransfer, GMP would be transferred to newly synthesized ppRNA (see introduction). To date, however, there are no available data fully proving the transfer of GMP on a ppRNA substrate catalyzed by a flavivirus NS5MTase domain.

#### 3.2.2 NS5 RNA-dependent RNA polymerase domain

The first structure of a flavivirus NS5RdRp domain KUNV NS5RdRp was determined within the VIZIER Project (Malet et al., 2007). The low sequence identity of flavivirus RdRps compared to other RdRps with existing structures (Ferrer-Orta et al., 2005) precluded the use of the molecular replacement method. Starting from a number of constructs of the KUNV NS5RdRp domain comprising residues 273–905 with N- and C-terminal deletions, the structure of a shorter form (317–905) could be solved using single anomalous dispersion at 2.35 Å resolution. This form of KUNV NS5RdRp then allowed structure determination of the longer form (273–905) at 3.0 Å resolution. The longer form was enzymatically active whereas the shorter form was not (Malet et al., 2007). The KUNV NS5RdRp structure then allowed the determination of the structure of the NS5RdRp domain (starting at residue 273) of DENV3 NS5RdRp at 1.85 Å resolution by molecular replacement (Yap et al., 2007).

##### 3.2.2.1 General structural properties of the polymerase domain

**Fig. 10:**
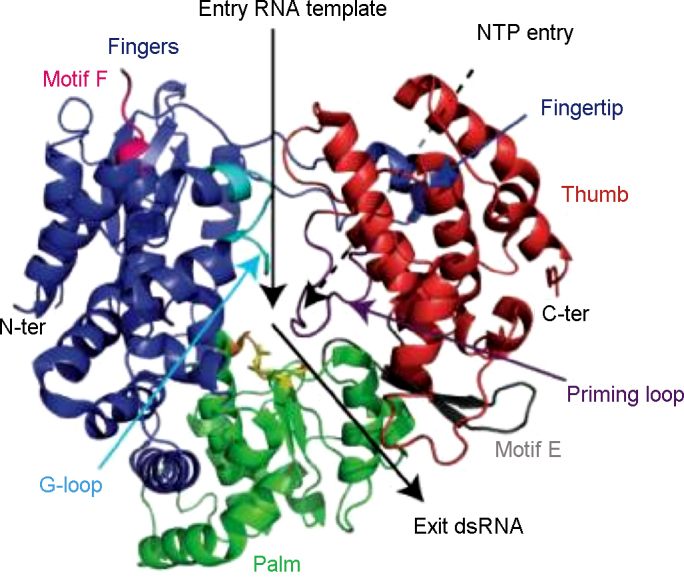
Overview of the flavivirus RdRp structure based on WNV NS5Pol (Malet et al., 2007) as an example; a “Front” view is presented here in ribbon representation. Fingers, palm and thumb subdomains are colored in blue, green and red, respectively. The ssRNA template entry and the dsRNA exit are shown by black arrows. A dotted arrow points to the NTP entry tunnel at the back of the RdRp. Motifs A, C, E, F, the G-loop and the priming loop are colored in orange, yellow, grey, magenta, cyan and purple, respectively. The Asp residues of catalytic motifs A and C (Asp-533, Asp-663 and Asp-664) are represented as stick models. N-ter and C-ter indicate the termini of the RdRp domain.

The structure of the two flavivirus NS5RdRp domains have been recently analyzed and reviewed in (Malet et al., 2008). They adopt a typical RdRp right-hand structure comprising three subdomains: fingers, palm and thumb (Fig. 10). The fingers subdomain of the short KUNV NS5RdRp construct was partially disordered, whereas the long constructs started with the ordered N-terminal helix (277–287 and 275–285 for KUNV NS5RdRp and DENV NS5RdRp, respectively). Both flavivirus NS5RdRp domains display a closed conformation, where fingers and thumb subdomains are connected. This is a characteristic of RdRps and in particular of primer-independent (de novo) RdRps (reviewed in Ferrer-Orta et al., 2005). A structural element named the priming loop provides the initiation platform. It belongs to the thumb subdomain and points towards the active site in the palm. The active site is located at the intersection of two tunnels. Other de novo RdRps solved in complex with ssRNA template, NTPs and/or dsRNA product (Butcher et al., 2001, Tao et al., 2002, O’Farrell et al., 2003) suggest the following scenario shown in Fig. 10. The first tunnel, located between the fingers and the thumb, should allow the ssRNA template to access the active site. The second tunnel, roughly perpendicular to the first, stretches across the entire protein. The incoming NTP should arrive from the back of the tunnel and, after the polymerization has started, the nascent dsRNA should go out through the front of this tunnel. However, as for other de novo RdRps, a conformational change is necessary to avoid a steric clash with the priming loop and allow neo-synthesized RNA to exit.

**Fig. 11:**
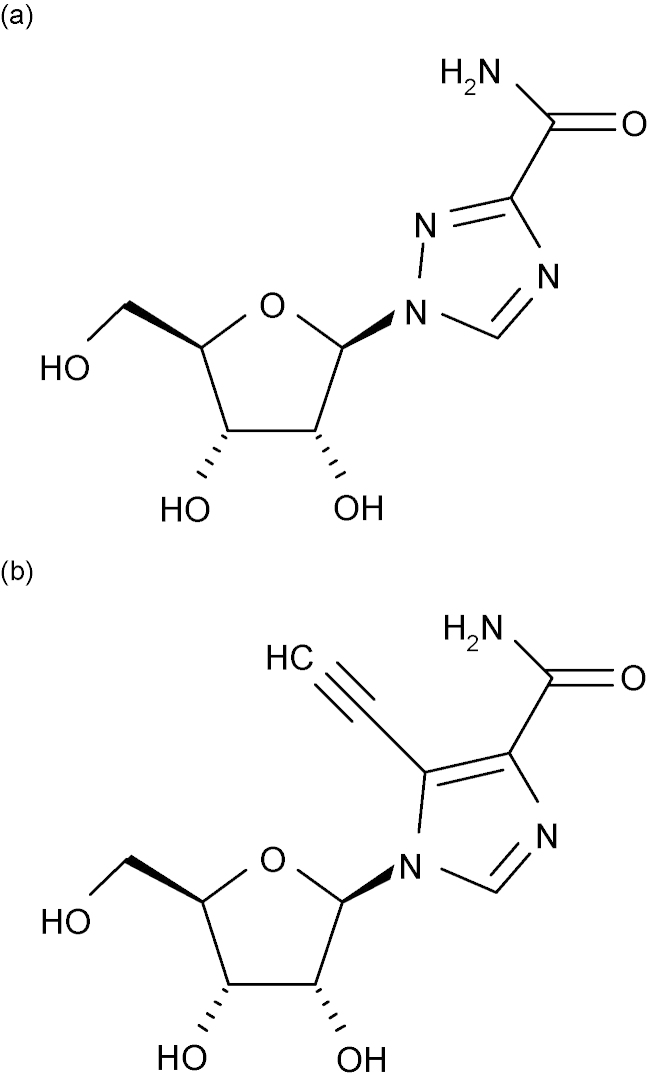
Structural formulae of (a) ribavirin and (b) EICAR.

As expected, the most closely related structures are those of de novo RdRps from members of the Flaviviridae family, namely HCV NS5RdRp (Ago et al., 1999, Bressanelli et al., 1999, Lesburg et al., 1999) and bovine viral diarrhea virus (BVDV) NS5RdRp (Choi et al., 2004). They show sequence identities as low as 11–21%, depending on the subdomain considered (Malet et al., 2007). Special features of flavivirus NS5RdRp structures are as follows (Malet et al., 2008): (1) The fingers subdomain was captured in a pre-initiation conformation since motif F, normally comprising the NTP-binding sites does not form the upper part of the NTP tunnel but is perpendicular to its normal position and partially disordered (Fig. 11). Additionally, the fingers subdomain presents a loop, named G-loop, which protrudes towards the active site (see Fig. 11). It was given the name G-loop because it harbours RdRp motif G in primer-dependent RdRps (Gorbalenya et al., 2002, Ferrer-Orta et al., 2004). This loop may play a regulatory role similar to the C-terminal in other RdRps (Adachi et al., 2002, Leveque et al., 2003, Ng and Parra, 2004). In summary, a concerted conformational change of motif F and G-loop of the trapped pre-initiation conformation is expected before flavivirus NS5RdRp initiate RNA synthesis. (2) The priming loop is provided by the thumb domain as observed for other de novo RdRps (bacteriophage phi6, HCV and BVDV RdRps (Ago et al., 1999, Bressanelli et al., 1999, Lesburg et al., 1999, Butcher et al., 2001, Choi et al., 2004) but does not contain any secondary structure. Two aromatic residues Trp795 or His798 (DENV NS5RdRp), may act as initiation platform stacking with the priming nucleotide. (3) Two Zn ions were found, one in the fingers and one in the thumb subdomain. The latter is localized at a supposed hinge position between the thumb and the palm subdomains. It might play a role in the regulation of the conformational change between initiation and elongation state of flavivirus NS5RdRps. (4) Two NLSs are present at the surface of the fingers subdomain. They comprise the first fingertip forming the connection between finger and thumb domain (see Fig. 11), run across the back of the NS5RdRp domain and end in an α-helix, which comes to the front and forms the interface between the bottom of the fingers subdomain and the palm.

The NLS of DENV2 NS5 has been shown to be functional and the transport of NS5 to the nucleus vital for virus replication (Pryor et al., 2007). Especially two lysine residues at the beginning of the α-helix seem to be important. In contrast, KUNV NS5 does not localize to the nucleus. Subtle differences in NLS geometry and charge distribution may be responsible for distinct behavior towards nuclear import in closely related viruses, but this is not yet deducible from the structures. It has not been shown if NS5 of DENV3 localizes to the nucleus (Malet et al., 2008).

##### 3.2.2.2 Three-dimensional structures determined for the flaviviral polymerase domain

Two flavivirus NS5RdRp domain structures are known so far, KUNV NS5RdRp and DENV3 NS5RdRp (Malet et al., 2007, Yap et al., 2007). The structures are very similar with an r.m.s.d. of 1.9, 0.8 and 1.0 Å (Cα atoms of matched residues) for the fingers, palm and thumb subdomains, respectively. The overall r.m.s.d. is expected to be high because of a domain rotation based on the hinge region between the thumb and the palm subdomains near the Zn atom (see above). The rotation of the fingers subdomain by 8° leaves the DENV3 NS5RdRp structure more open in comparison to KUNV NS5RdRp. Another consequence is that the active site of KUNV NS5RdRp is more tightly closed by the priming loop than the active site of DENV3 NS5RdRp. KUNV NS5RdRp may need a wider opening movement upon the transition to elongation mode, which could explain why the transition from initiation to elongation seems to be kinetically more limiting for KUNV NS5RdRp compared to DENV2 NS5RdRp (Selisko et al., 2006).

Both flavivirus NS5RdRp structures were obtained with a Mg^2+^ ion in a non-catalytic position near the active center. The role of the non-catalytic ion is not known. It was proposed that it might play a role in the de novo initiation mechanism facilitating the movement of the nascent dsRNA after formation of the first dinucleotide out of the active site (Butcher et al., 2001). DENV3 NS5RdRp crystals were soaked with the nucleotide analog 3′dGTP and the complex structure solved at 2.6 Å resolution. Only the triphosphate moiety of 3′dGTP is visible. Nevertheless, its position near the priming loop and in particular next to residue Trp795 lead to the proposal that it represents the priming nucleotide and Trp795 acts as the initiation platform (Yap et al., 2007). This is consistent with a model of the de novo initiation complex of KUNV NS5RdRp, which assigned the same Trp, conserved in all flavivirus NS5 proteins, as initiation platform (Malet et al., 2007).

##### 3.2.2.3 Characterization of polymerase activity

Within the VIZIER Project, a comparison of steady-state enzymatic activity parameters of both full-length NS5 and NS5RdRp domain of DENV2 and KUNV on a homopolymeric template poly(rC) suggested that the NS5MTase domain does not influence de novo RdRp activity (Selisko et al., 2006, Zhang et al., 2008), although others did not support fully this view (Yap et al., 2007). The NS5RdRp domain alone was used for screening processes and characterization of inhibitors of the flavivirus NS5RdRp activity. Furthermore, it has been shown by atomic force microcopy that a NS5RdRp domain of DENV2 with the same boundaries (272–900) binds to the circularized DENV2 RNA genome and that de novo RNA synthesis of the negative strand is enhanced by the presence of a promoter element, a large stem-loop structure, named SLA, present at the 5′-end of the genome (Filomatori et al., 2006). The authors demonstrated the physical interaction of the NS5RdRp domain with SLA. They proposed a novel mechanism for −ssRNA synthesis in which the flavivirus NS5RdRp is recruited by and specifically binds SLA at the 5′-end of the genome. It then reaches the site of initiation at the 3′-end recruited to the 5′-end via long-range RNA–RNA interactions.

The VIZIER Project contributed decisively to a precise identification of the flaviviral NS5RdRp domain and to its subsequent structural characterization (Selisko et al., 2006, Malet et al., 2007, Yap et al., 2007). Our contributions will greatly facilitate the exploration of the flavivirus NS5RdRp as a drug target (Rawlinson et al., 2006, Malet et al., 2008), hopefully leading to the discovery and design of drugs against flaviviruses.

## 4 Antivirals

A safe and efficient anti-flavivirus/anti-DENV drug could potentially be used for the treatment of patients living in endemic regions and presenting symptoms of DENV infection, as well as patients with laboratory-diagnosed DENV infection. Such drug may also be of prophylactic relevance in case of an epidemic, in particular in regions where more than one serotype is circulating. Another possible prophylactic use can be by travelers to and through endemic regions and by personnel of NGOs working in endemic regions or by military personnel carrying out humanitarian actions. Because severe DENV disease has been associated with higher virus titres (Whitehead et al., 2007), reduction of viral replication may be instrumental to limit the risk of developing such symptoms.

The ideal flavivirus drug should preferably be active against all four DENV serotypes (and even other flavivirus infections, such as WNV and JEV). Such drug should be (i) administered via the oral route and should thus have high oral bioavailability. Ideally, it should be (ii) administered only once or twice (maximum 3 times) daily and (iii) have a high genetic barrier to resistance. Obviously, (iv) a drug that is to be used in the prophylactic setting as well as in pediatric patients should be very safe. Moreover, since flavivirus drugs will be used in tropical regions, the drug should also have (v) good thermal stability and a good hygroscopic parameter (high temperature and level of humidity). Moreover, (vi) the production should be “easy” and low-cost (limited number of chemical steps and common availability of the starting material).

### 4.1 A broad-spectrum antiviral molecule with weak activity

Ribavirin (1-beta-d-ribofuranosyl-1,2,4-triazole-3-carboxamide, Fig. 11a) is a broad-spectrum inhibitor of RNA viruses replication proved to treat HCV infections, in combination with pegylated interferon and in aerosol form, for the treatment of pediatric respiratory syncytial virus (RSV) infections. Ribavirin has also been used experimentally against a number of other conditions, including Lassa fever, Crimean-Congo hemorrhagic fever virus (CCHFV), and hantaviruses (Ergonul, 2008, Jonsson et al., 2008, Khan et al., 2008). Almost all RNA viruses and even some DNA viruses are sensitive to the *in vitro* antiviral activity of ribavirin. Some viruses are more susceptible to the action of ribavirin than others; flaviviruses, for example, are much less sensitive than the paramyxovirus RSV (Leyssen et al., 2005).

**Fig. 12:**
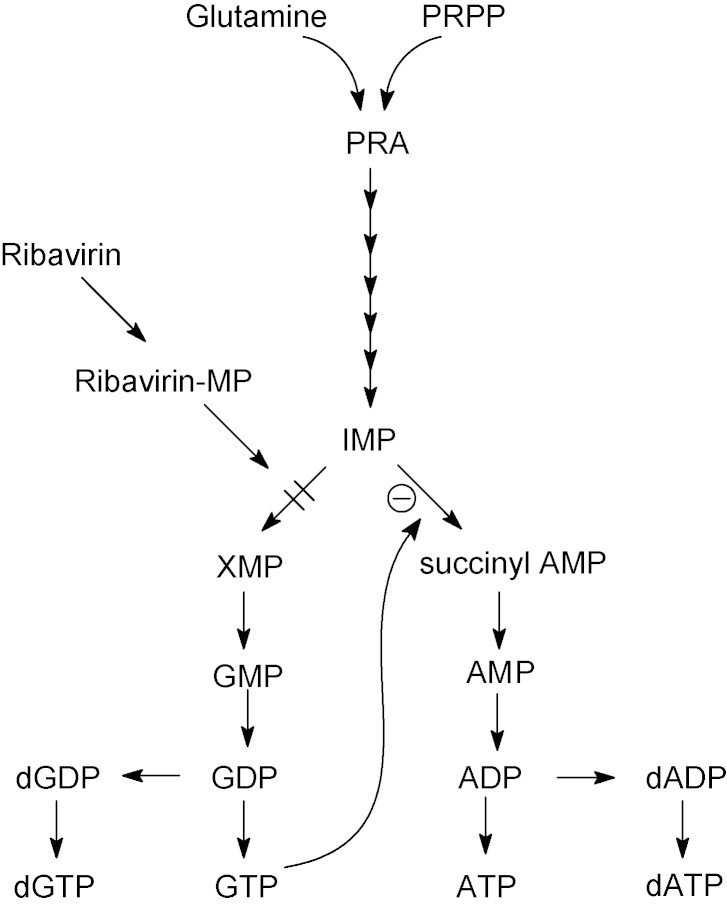
Mechanism of ribavirin action. Target enzyme: IMP dehydrogenase. Ribavirin 5′ monophosphate inhibits the conversion of IMP to XMP resulting in a reduced supply of GTP, and, indirectly, also a reduced supply of ATP.

The antiviral activity of ribavirin was reported almost four decades ago, but the molecular mechanism by which the compound exerts its antiviral activity still remains a matter of debate. Inosine 5′-monophosphate (IMP) dehydrogenase, a cellular enzyme which converts IMP to xanthosine 5′-monophosphate in the *de novo* synthesis pathway of GMP, is inhibited by ribavirin 5′-monophosphate (Streeter et al., 1973). As a consequence, intracellular GTP pools are depleted, resulting in inhibition of viral (but also cellular) RNA synthesis (Fig. 12). Several other mechanisms have been proposed to contribute to the antiviral activity of ribavirin, including inhibition of viral capping (via an effect on the viral GTase or MTase activities) (Benarroch et al., 2004, Bougie and Bisaillon, 2004), and inhibition of RdRp activity by the 5′-triphosphate group of the drug (Maag et al., 2001, Bougie and Bisaillon, 2003). Moreover, inhibition of the viral helicase activity by ribavirin has been proposed for reoviruses (Rankin et al., 1989).

Poliovirus cultured in the presence of ribavirin (concentrations up to 1 mM) accumulated mutations in its genome, a process called error catastrophe (Crotty et al., 2001). For flaviviruses we demonstrated that GTP pool depletion is the predominant mechanism by which ribavirin exerts its antiviral activity (Leyssen et al., 2005) and that an error catastrophe based mechanism does not contribute to the *in vitro* antiviral activity of the drug (Leyssen et al., 2006). The effect of ribavirin was studied in rhesus monkeys infected with YFV or DENV1. Either therapeutic or prophylactic protocols were studied. Overall, no effect on viremia and survival was noted (Huggins et al., 1984, Huggins, 1989, Malinoski et al., 1990). Since the mechanism of anti-flavivirus activity of ribavirin is based on an aspecific mechanism, the design of safe and more potent analogues of ribavirin will likely be very difficult to achieve. EICAR (Fig. 11b), the 5-ethynyl analogue of ribavirin, was shown to be roughly 10–20-fold more potent in inhibiting flaviviruses replication *in vitro*. This improved activity came, however, at the price of a concomitant increase in toxicity (Leyssen et al., 2005), which is explained by the fact that EICAR 5′-monophosphate is also more potent in inhibiting the IMP dehydrogenase (Balzarini et al., 1993). Recently, a heterocyclic molecule with *in vitro* anti-DENV activity was reported; the mechanism of action was suggested to be related to the inhibition of cellular IMP dehydrogenase (Nair et al., 2009).

### 4.2 Selective inhibitors of viral replication

**Fig. 13:**
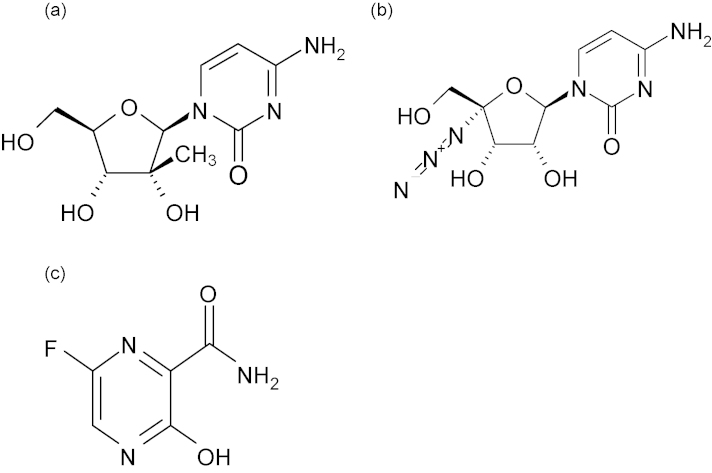
Structural formulae of (a) 2′-c-methylcytidine, (b) 4′-azidocytidine and (c) T-705.

The HCV RdRp has been shown to be an excellent target for inhibition of viral replication. In fact, numerous selective inhibitors of HCV replication that target this enzyme have been identified so far. These compounds can largely been classified as nucleoside (that need to be phosphorylated to their 5′-triphosphate metabolite) and non-nucleoside inhibitors (that act as allosteric site inhibitors) (De Clercq and Neyts, 2009). The nucleoside HCV inhibitors 2′-C-methylcytidine (Fig. 13a), and related 2′-C-methyl nucleosides, inhibit the replication of a broad spectrum of (+)ssRNA viruses including flaviviruses (Eldrup et al., 2004). Another nucleoside inhibitor, i.e. 4′-azidocytidine (Fig. 13b), is solely active against HCV and does not show activity against flaviviruses and other RNA viruses (Klumpp et al., 2006, and unpublished data). It remains to be studied what is the structural basis for the broad-spectrum activity versus RNA viruses of the 2′-C-methyl nucleoside analogues and the lack of activity of the 4′-azido nucleoside analogues.

Recently, a substituted pyrazine (T705, Fig. 13c) has been reported to be a potent inhibitor against influenza A, B and C viruses *in vitro* (Furuta et al., 2002, Furuta et al., 2005). It has been proposed that T-705 is converted intracellularly to the ribonucleotide T-705-ribofuranosyl-5′-monophosphate (T-705 RMP) by a phosphoribosyl transferase, and, upon phosphorylation, to its 5′-triphosphate. This metabolite would inhibit the influenza virus RdRp in a GTP-competitive manner (Furuta et al., 2002, Furuta et al., 2005). Unlike ribavirin 5′-monophosphate, T-705 RMP does not significantly inhibit IMP dehydrogenase, indicating that it may owe its anti-influenza virus activity mainly, if not exclusively to inhibition of the influenza virus RNA polymerase. Surprisingly, T-705 has also been accredited with both activity against other viruses, i.e. arenaviruses (Pichinde), and bunyaviruses (Punta Toro) and flaviviruses. In addition to inhibiting YFV and WNV replication *in vitro*, improvements in survival and disease parameters were observed also after addition of T-705 to YFV- or WNV-infected rodents (Morrey et al., 2008, Julander et al., 2009). It may be assumed that the mechanism by which T-705 inhibits viruses other than influenza is similar to the mechanism by which it is believed to inhibit influenza virus replication. This remains subject of further studies. Such studies may also provide insight on how broad-spectrum inhibitors of RNA viruses encompassing both (−)ssRNA and (+)ssRNA viruses should be designed,.

So far, non-nucleoside inhibitors of flavivirus replication which target the viral NS5RdRp domain have not yet been reported. Within the VIZIER Project, potential allosteric inhibitor binding sites were predicted on the NS5RdRp of DENV and WNV, using two different programs (Malet et al., 2008). Since several classes of non-nucleoside inhibitors of pestiviruses and HCV RdRp (and four allosteric binding sites) have been identified, it may be assumed that this class of inhibitors may also have potential against flaviviruses.

### 4.3 Identification of novel antivirals

As outlined above, target-based design of inhibitors of flavivirus replication may be a promising strategy towards the development of selective anti-flaviviral drugs. Another strategy, with a proven success in the development of inhibitors of for example HIV, herpes and HCV, has been based on the screening of large libraries of molecules. Infected-cell-based screening assays offer the advantage that (i) novel targets for antiviral therapy in the replication cycle of the virus that would not have been discovered in target-based assays may be identified, and (ii) compounds that do not enter the host cell or that are toxic to the host cell will be excluded for further validation. Several examples of antiviral drugs that would not have been identified in target-based screenings assays include (i) the BVDV non-nucleoside inhibitors (VP32947, BPIP, AG110, LZ37) that target the viral RdRp, but that do not exhibit inhibitory activity on the purified RdRp, (ii) the imidazopyridines with anti-HCV activity, of which one analogue (GS-9190), targeting the HCV RdRp but not inhibiting the activity of the purified enzyme, is currently in phase II clinical studies (Vliegen et al., 2009), and (iii) the cyclophilin-binding agent Debio-025, a potent inhibitor of HCV replication (currently in phase II clinical studies) that prevents HCV replication by interfering with cyclophilins (which are essential in the replication cycle of HCV), but that has not been shown to directly inhibit a particular enzymatic function of the virus (Chatterji et al., 2009, Coelmont et al., 2009).

#### 4.3.1 High-throughput screening approach

Most flaviviruses that are pathogenic to humans, readily cause a cyto-pathogenic effect (CPE) in cell culture, being thus amenable to high-throughput screening programs. Compounds to be screened should not contain potential toxic functions, should not have unstable chemical groups, or poor solubility, and should ideally comply with Lipinski's rule of five or the Veber rules. Furthermore, the library can be enriched for drug-like compounds (Comprehensive Medicinal Chemistry mapping). To identify molecules with potential *in vitro* anti-DENV activity (hit compounds), a primary screen is run (using multiple dilutions/compound to exclude too many false positives) and the potential inhibitory effect on virus-induced CPE is quantified [employing a luminescence-based metabolic assay (ATP-lite)]. Concomitantly with the evaluation of the antiviral effect, the potential anti-metabolic effect of the compounds on uninfected cells is quantified [employing an absorbance-based metabolic assay]. Once hit compounds have been identified, the antiviral activity and selectivity need to be confirmed using a newly synthesized batch of the molecule. Next, the antiviral activity, either based on quantifying the effect of the compound on the infectious virus-yield and/or the effect on viral RNA production, is confirmed in virus-yield assays. Once this has been accomplished, a hit-to-lead optimization process can be initiated, given the fact that the compound class is chemically tractable. Meanwhile studies to characterize the antiviral activity and to identify the molecular target are performed.

Within the VIZIER consortium, two compound classes with antiflavivirus activity were identified. A first compound, SA-17, identified as a potent inhibitor of flavivirus replication, is an analogue of doxorubicin (an antineoplastic antibiotic from Streptomyces peucetius) that carries a squaric acid amide-ester moiety at the carbohydrate (α-l-daunosaminyl) of doxorubicin. It should be mentioned that this molecule does not comply with the Lipinski rule of five. SA-17 was found to have excellent activity against DENV (EC50 = 0.3 μg/ml) and is markedly less cytostatic than the parent compound (CC50 = 28 μg/ml). SA-17 also inhibited YFV-17D replication, although less efficiently than DENV replication, but proved inactive against other viruses (the picornavirus coxsackievirus B3, the retroviruses HIV-1 and HIV-2, and the herpesvirus HSV-1). SA-17 inhibits flavivirus replication in Vero cells in a dose-dependent manner, as assessed by virus-yield reduction assays and quantification of viral RNA by means of q-RT-PCR. The anti-DENV activity was confirmed using a Renilla luciferase expressing DENV reporter. Time-of-drug addition studies revealed that SA-17 acts at the very early stages of the viral replication cycle. This observation was corroborated by the observation that SA-17, unlike the nucleoside analogue ribavirin, does not inhibit the replication of DENV subgenomic replicons. Preincubation of high-titre DENV or YFV-17D stocks with 5 or 10 μg/ml SA-17 for 1 h resulted in 100% inhibition of viral infectivity. Inhibition of viral infectivity by SA-17 in such pre-incubation experiments correlates with the antiviral effect obtained in virus-yield assays. Molecular modeling studies identified a putative binding site for SA-17 in the DENV glycoprotein E (Kaptein et al., submitted for publication).

A second compound class, identified in a large screening effort, consists of a series of small drug-like molecules that inhibit the replication of both YFV and DENV2 replication (representative molecule CHI-104). Unlike SA-17, it appeared from time of drug addition studies that this class of compounds interferes with a step in the replication cycle of flaviviruses that coincides with the onset of viral RNA synthesis. This was corroborated by the observation that the compounds are active (like the reference compound ribavirin, but unlike compound SA-17 that acts at an early stage of the replication cycle) in the DENV subgenomic replicon system. Currently, drug-resistant variants against CHI-104 (and analogues) are being generated. It is expected that drug-resistant variants will carry mutations in the NS genes encoded by the replicon. Reintroduction of the mutations identified in drug-resistant viruses in the wild-type genome should result in a resistant phenotype. Once the target has been identified using this approach, the gene of interest will be expressed and, if an enzymatic activity is associated with this protein, a potential inhibitory activity of the compound (class) on this protein will be studied. If a crystal structure of the target protein is available, this should allow to define (based on soaking or co-crystallization experiments with subsequent complex structure determination) the precise molecular interactions between the inhibitor and the target. In turn, this will allow a rational approach to optimize the antiviral activity/selectivity.

#### 4.3.2 Virtual docking of small molecules

In the context of the VIZIER Project, about one hundred crystal structures of important enzymes relevant for viral replication have been determined. The 3D structure of an enzyme, and, in particular, of its active site, can be an useful tool to identify possible inhibitors of the target protein. In virtual docking, a library of small molecules is used to identify ligands with high binding affinity to the protein active site. In VIZIER Project, in order to find new flavivirus inhibitors, many efforts have been spent on the study of the NS5MTase enzyme. In particular, the binding site of its cofactor AdoMet was used to screen a set of 7836 potential ligand structures by virtual docking (Luzhkov et al., 2007). The structures were generated by geometry optimization and ligand preparation of 2566 hits that had been selected from a data base of 2.1 million commercially available compounds after conducting a pharmacophore and a 2D similarity search. One of the top binders was found to inhibit the 2′OMTase activity of DENV NS5MTase with an IC_50_ value of 60.5 μM. Another approach for NS5MTase inhibition was based on the mechanism of action of the enzyme. In this case, the virtual search was directed to find molecules potentially able to bind to the protein active site in presence of the AdoMet cofactor. In the latter approach, it was chosen not to interfere with the binding of the cofactor to the protein but with the methyl transfer activity (Milani et al., 2009). Therefore, virtual screening of a compounds library yielded a ligand capable to bind to the NS5MTase active site in presence of AdoMet. Such a ligand can interfere with the methyl transfer activity of the enzyme because of steric hindrance in the active site. In both cases, inhibition of AdoMet binding site or hindrance of the enzyme active site, the two molecules identified showed activity against the enzyme (N7MTase and 2′OMTase activities). The potential use of the two new inhibitors against viral replication is still under investigation.

In addition to the components of the flaviviral replicase complex, the protease is also of high interest as a target for new antivirals (see Section 3.1.1 above). Here, some lessons can be learned from drug discovery efforts performed in order to inhibit the proteases of HCV and HIV, both well established drug targets. As various high-throughput screening attempts did not produce the originally sought results, researchers chose structure-based design strategies to develop potent inhibitors. This resulted in impressive success stories (Anderson et al., 2009, Lamarre et al., 2003, Tsantrizos, 2004). Similar strategies are currently being applied to the flavivirus NS2B/NS3 proteases, not only in academic laboratories, but increasingly also in the pharmaceutical industry.
