# Supplementary material for: Structure and functionality in flavivirus NS-proteins: Perspectives for drug design
Source: Antiviral Res. 2010 Aug;87(2):125–48. doi: 10.1016/j.antiviral.2009.11.009 (PMC3918146; doi:10.1016/j.antiviral.2009.11.009)
Supplement: Supplementary file 1 [file mmc1.doc]

Table 1: Flaviviral abbreviation

| **Tick-borne viruses** | **TBVs** |
| --- | --- |
| Gadget Gully virus | GGYV |
| Kadam virus | KADV |
| Kyasanur Forrest disease virus | KFDV |
| Langat virus | LGTV |
| Omsk hemorrhagic fever virus | OHFV |
| Powassan virus | POWV |
| Royal farm virus | RFV |
| Karshi virus | KSIV |
| tick-borne encephalitis virus | TBEV |
| Louping ill virus | LIV |
| Meaban virus | MEAV |
| Saumarez Reef virus | SREV |
| Tyuleniy virus | TYUV |
| Ngoye virus | NGOV |
|  |  |
| **Mosquito-borne viruses** | **MBVs** |
| Aroa virus | AROAV |
| Bussuquara virus | BSQV |
| Iguape virus | IGUV |
| Naranjal virus | NJLV |
| Dengue virus | DENV |
| Kedougou virus | KEDV |
| Cacipacore virus | CPCV |
| Koutango virus | KOUV |
| Japanese encephalitis virus | JEV |
| Murray Valley encephalitis virus | MVEV |
| Alfuy virus | ALFV |
| St Louis encephalitis virus | SLEV |
| Usutu virus | USUV |
| West Nile virus | WNV |
| Kunjin virus | KUNV |
| Yaounde virus | YAOV |
| Kokobera virus | KOKV |
| Stratford virus | STRV |
| Bagaza virus | BAGV |
| Ilheus virus | ILHV |
| Rocio virus | ROC |
| Israel turkey meningoencephalomyelitis virus | ITV |
| Ntaya virus | NTAV |
| Tembuzu virus | TMUV |
| Zika virus | ZIKV |
| Spondweni virus | SPOV |
| Banzi virus | BANV |
| Bouboui virus | BOUV |
| Edge Hill virus | EHV |
| Jugra virus | JUGV |
| Potiskum virus | POTV |
| Saboya virus | SABV |
| Sepik virus | SEPV |
| Uganda S virus | UGSV |
| Sitiawan virus | SV |
| Kamiti River virus | KRV |
| Wesselsbron virus | WESSV |
| yellow fever virus | YFV |
| Nounané virus | NOUV |
| Barkedji virus |  |
|  |  |
| **Viruses with no known arthropod vector** | **NKVs** |
| Entebbe bat virus | ENTV |
| Sokoluk virus | SOKV |
| Yokose virus | YOKV |
| Apoi virus | APOIV |
| Cowbone Ridge virus | CRV |
| Jutiapa virus | JUTV |
| Modoc virus | MODV |
| Sal Vieja virus | SVV |
| San Perlita virus | SPV |
| Bukalasa bat virus | BBV |
| Carey Island virus | CIV |
| Dakar bat virus | DAKV |
| Montana Myotis leukoencephalitis virus | MMLV |
| Phnom Penh bat virus | PPBV |
| Batu Cave virus | BCV |
| Rio Bravo virus | RBV |
| cell fusing agent virus | CFAV |
| Tamana bat virus | TABV |
