# Supplementary material for: Structure and functionality in flavivirus NS-proteins: Perspectives for drug design
Source: Antiviral Res. 2010 Aug;87(2):125–48. doi: 10.1016/j.antiviral.2009.11.009 (PMC3918146; doi:10.1016/j.antiviral.2009.11.009)
Supplement: Supplementary file 2 [file mmc2.doc]

Table 2: Overview about the currently PDB-deposited crystal structures of Flavivirus NS3 proteases (April 2009)

| **Viral Protease** | **Resolution [Å]** | **Ligand/Inhibitor** | **PDB entry** | **Reference, year of publication** |
| --- | --- | --- | --- | --- |
| DV2 NS3pro | 2.1 | uncomplexed | 1BEF | Murthy et al., 1999 |
| DV2 NS3pro | 2.1 | Mung Bean Bowman-Birk inhibitor | 1DF9 | Murthy et al., 2000 |
| DV2 NS3pro | 2.1 | Mung Bean Bowman-Birk inhibitor | 2QID | Murthy et al., To be published |
| DV4 NS2b/NS3 protease-helicase | 3.15 | uncomplexed | 2VBC | Luo et al., 2008 |
| DV2 NS2B/NS3pro | 1.5 | uncomplexed | 2FOM | Erbel et al., 2006 |
| WNV NS2B/NS3pro | 1.68 | Covalently bound peptide-type inhibitor | 2FP7 | Erbel et al., 2006 |
| WNV NS2B/NS3pro, His51Ala mutant | 1.8 | uncomplexed | 2GGV | Aleshin et al., 2007 |
| WNV NS2B/NS3pro | 2.3 | aprotinin | 2IJO | Aleshin et al., 2007 |
| WNV NS2B/NS3pro | 2.45 | Covalently bound peptide-type inhibitor | 3E90 | Robin et al., 2009 |
