# Supplementary material for: Structure and functionality in flavivirus NS-proteins: Perspectives for drug design
Source: Antiviral Res. 2010 Aug;87(2):125–48. doi: 10.1016/j.antiviral.2009.11.009 (PMC3918146; doi:10.1016/j.antiviral.2009.11.009)
Supplement: Supplementary file 3 [file mmc3.doc]

Table 3: Flaviviral MTases crystal structures

| Virus | PDB ID | Ligand(s) | Reference | Vizier |
| --- | --- | --- | --- | --- |
| WESSV | 3ELY | AdoHcy | Bollati et al., 2009a | Yes |
|  | 3ELU | AdoMet | Bollati et al., 2009a | Yes |
|  | 3ELW | AdoMet + GpppG | Bollati et al., 2009a | Yes |
|  | 3EMB | AdoMet + N7MeGpppG | Bollati et al., 2009a | Yes |
|  | 3ELD | Sinefungin | Bollati et al., 2009a | Yes |
|  | 3EMD | Sinefungin + N7MeGpppA | Bollati et al., 2009a | Yes |
| MVEV | 2PX2 | AdoHcy | Assenberg et al., 2007 | Yes |
|  | 2PX4 | AdoHcy | Assenberg et al., 2007 | Yes |
|  | 2PX5 | AdoHcy | Assenberg et al., 2007 | Yes |
|  | 2PXC | AdoMet + GpppA | Assenberg et al., 2007 | Yes |
|  | 2PX8 | AdoHcy + N7MeGTP | Assenberg et al., 2007 | Yes |
|  | 2PXA | AdoHcy + GpppG | Assenberg et al., 2007 | Yes |
| DENV | 1L9K | AdoHcy | Egloff et al, 2002 | No |
|  | 3EVG | AdoHcy | Geiss et al., 2009 | No |
|  | 1L9K | AdoHcy + Ribavirin triphosphate | Egloff et al, 2002 | No |
|  | 2P41 | AdoHcy + N7MeGpppG2'OMe | Egloff et al, 2007 | Yes |
|  | 2P40 | AdoHcy + N7MeGpppG | Egloff et al, 2007 | Yes |
|  | 2P3Q | AdoHcy + GpppG | Egloff et al, 2007 | Yes |
|  | 2P3O | AdoHcy + N7MeGpppA | Egloff et al, 2007 | Yes |
|  | 2P3L | AdoHcy + GpppA | Egloff et al, 2007 | Yes |
| YFV | 3EVA | AdoHcy | Geiss et al., 2009 | No |
|  | 3EVB | AdoHcy | Geiss et al., 2009 | No |
|  | 3EVC | AdoHcy + GTP | Geiss et al., 2009 | No |
|  | 3EVD | AdoHcy + GTP | Geiss et al., 2009 | No |
|  | 3EVE | AdoHcy + GpppA | Geiss et al., 2009 | No |
|  | 3EVF | AdoHcy + N7MeGpppA | Geiss et al., 2009 | No |
| WNV | 2OY0 | AdoHcy | Zhou et al., 2007 | No |
| MEAV | 2OXT | AdoMet | Mastrangelo et al., 2007 | Yes |
| YOKV | 3GCZ | AdoMet | Bollati et al., 2009b | Yes |
